# Application of recombinant antibodies for treatment of *Clostridioides difficile* infection: Current status and future perspective

**DOI:** 10.3389/fimmu.2022.972930

**Published:** 2022-08-23

**Authors:** Hamideh Raeisi, Masoumeh Azimirad, Ali Nabavi-Rad, Hamid Asadzadeh Aghdaei, Abbas Yadegar, Mohammad Reza Zali

**Affiliations:** ^1^ Foodborne and Waterborne Diseases Research Center, Research Institute for Gastroenterology and Liver Diseases, Shahid Beheshti University of Medical Sciences, Tehran, Iran; ^2^ Basic and Molecular Epidemiology of Gastrointestinal Disorders Research Center, Research Institute for Gastroenterology and Liver Diseases, Shahid Beheshti University of Medical Sciences, Tehran, Iran; ^3^ Gastroenterology and Liver Diseases Research Center, Research Institute for Gastroenterology and Liver Diseases, Shahid Beheshti University of Medical Sciences, Tehran, Iran

**Keywords:** *Clostridioides difficile*, immunotherapy, hybridoma technology, recombinant antibodies, phage display

## Abstract

*Clostridioides difficile* (*C. difficile*), known as the major cause of antibiotic-associated diarrhea, is regarded as one of the most common healthcare-associated bacterial infections worldwide. Due to the emergence of hypervirulent strains, development of new therapeutic methods for *C. difficile* infection (CDI) has become crucially important. In this context, antibodies have been introduced as valuable tools in the research and clinical environments, as far as the effectiveness of antibody therapy for CDI was reported in several clinical investigations. Hence, production of high-performance antibodies for treatment of CDI would be precious. Traditional approaches of antibody generation are based on hybridoma technology. Today, application of *in vitro* technologies for generating recombinant antibodies, like phage display, is considered as an appropriate alternative to hybridoma technology. These techniques can circumvent the limitations of the immune system and they can be exploited for production of antibodies against different types of biomolecules in particular active toxins. Additionally, DNA encoding antibodies is directly accessible in *in vitro* technologies, which enables the application of antibody engineering in order to increase their sensitivity and specificity. Here, we review the application of antibodies for CDI treatment with an emphasis on recombinant fragment antibodies. Also, this review highlights the current and future prospects of the aforementioned approaches for antibody-mediated therapy of CDI.

## Introduction

**Figure 1:**
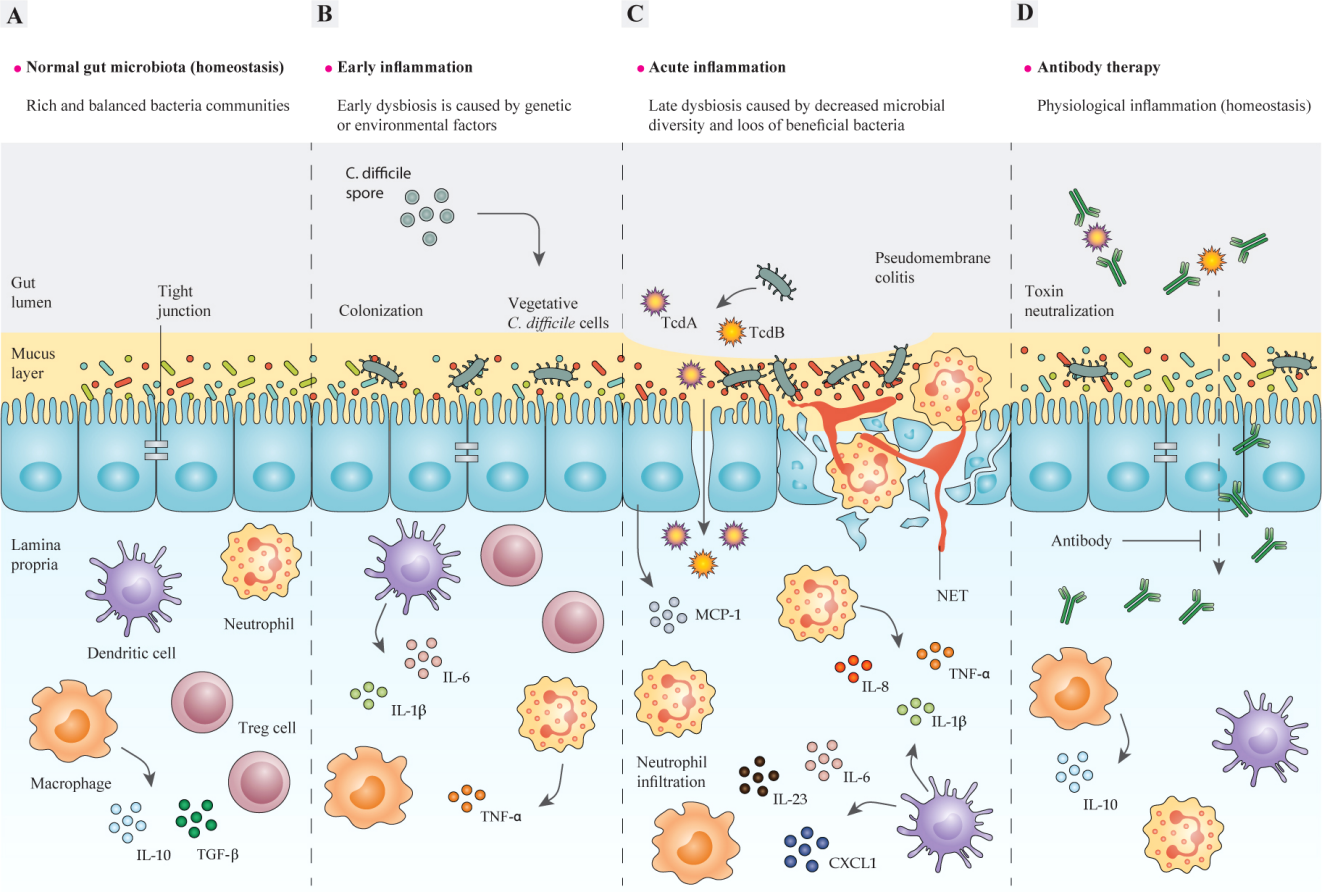
The structure of the intestinal epithelium in different stages of *C. difficile*-induced inflammation. **(A)** In the steady-state, intestinal bacteria are segregated from epithelial cells (IECs) by an intact mucosal layer. A well-balanced relationship is maintained between intestinal microbiota and mucosal barriers during gut homeostasis, so that gut microbes and host immune cells can mutually communicate to regulate the functions of intestinal epithelium. Commensal bacteria and pathogens can modulate the intestinal immune response to release various chemokines and cytokines, or inhibit their production, such as IL-8 and MCP-1. In a homeostasis state, pathogen recognition leads to inducing antigen-presenting cells (APCs), like macrophages, dendritic cells (DC), and neutrophils, which produce pro-inflammatory cytokines like IL-1β and IL-23. In contrast, commensals can stimulate APCs to promote anti-inflammatory cytokines, like IL-10 and Treg responses, which suppress the immune response by inhibiting cytokine production, therefore homeostasis and self-tolerance are maintained. In some cases, the interactions between commensal bacteria and the gut epithelium may lead to the discharge of TGF-β from macrophages, which triggers fibroblast proliferation (tissue remodeling). **(B)** The imbalance between mucosal barriers and gut microbes is promoted by the dysfunction of mucosal barriers, including decreased production of mucin that causes intestinal inflammation. A combination of genetic and environmental factors especially antibiotic administration, leads to gut microbiota dysbiosis, and thereby enrichment of pathobionts and susceptibility to *C. difficile* may occur. The adherence of *C. difficile* to the epithelium activates host inflammatory response via different signaling pathways, which result in production of inflammatory cytokines. **(C)** Epithelium colonization and toxin production by *C*. *difficile* act on colonic epithelial and immune cells as inflammatory stimuli and induce tissue damage. In particular, the cytopathic effects of TcdA and TcdB lead to disruption of the tight junctions, which causes toxins to cross the epithelial barriers and further induce inflammatory cytokine production in lymphocytes, macrophages, and DCs. This further contributes to inflammation and neutrophil influx, which subsequently results in a pseudomembrane formation, which is characteristic of *C*. *difficile* colitis. **(D)** The application of monoclonal antibodies (mAbs) developed for targeting toxins of *C. difficile* helps modulate direct damage to the colonic epithelium caused by toxins and restores the homeostatic immune responses and ameliorates inflammation. IECs, intestinal epithelial cells; IL-8, interleukin 8; MCP-1, monocyte chemoattractant protein-1; APCs, antigen-presenting cells; IL-1β, interleukin 1 beta; IL-23, interleukin 23; Treg, regulatory T cells; IL-10, interleukin 10; TGF-β, transforming growth factor-β; TNF-α, tumor necrosis factor alpha. IL-6, interleukin 6; DC, dendritic cell; TcdA, Toxin A; TcdB, Toxin B; mAbs, monoclonal antibodies.

*Clostridioides difficile*, formerly known as *Clostridium difficile*, is a Gram-positive species of spore-forming anaerobic bacteria and it can cause a range of different diseases in humans, such as antibiotic-associated diarrhea (AAD), pseudomembranous colitis (PMC), and toxic megacolon (1, 2). Additionally, *C. difficile* can asymptomatically colonize up to 3% of healthy adults (3). Normally, the bacterium is transmitted by shedding the spores in the environment and it can colonize the gastrointestinal (GI) tract of the infected patients where all of the aspects of *C. difficile* physiology are supported (4, 5). Currently, *C. difficile* infection (CDI) is known as the leading cause of nosocomial diseases associated with antibiotic therapy and healthcare-associated diarrhea in adults (6–8). CDI is characterized by neutrophil accumulation and appearance of distinct plaques (pseudomembranes) in the intestinal lumen (9, 10). It has been suggested that disturbance of the normal gut microbiota as a consequence of antibiotic treatment, could be regarded as a major promoting factor for CDI development (**Figures 1A, B**). Pathogenesis of *C. difficile* is based on the action of two pro-inflammatory toxins, TcdA and TcdB, that lead to cytoskeleton disintegration, condensation of actin, and eventually cell death (11, 12); these outcomes damage the patient’s colonic mucosa and cause severe diarrhea (**Figure 1C**).

Conventional CDI treatment includes antibiotic therapy that is universally prescribed, especially vancomycin or metronidazole, which are widely used for treating patients with CDI. These antibiotics are non-selective, thus there is a risk of further irritating gut dysbiosis (imbalance of microbiota) and reduction of normal gut commensals, favoring an appropriate niche for *C. difficile* to facilitate its colonization (13). Furthermore, recurrent CDI (rCDI) occurs in approximately 25% of patients and it becomes more prevalent and harder to treat after secondary and tertiary CDI (14, 15). The use of specific anti-*C. difficile* antibiotics, such as fidaxomicin, an antibiotic that has been approved by the Food and Drug Administration (FDA), is recommended for reducing the adverse effects of nonselective antibiotic therapy; for instance, fidaxomicin reduced the relapse rate compared to vancomycin, however, high costs of fidaxomicin restricted its application (16).

With the emergence of rising resistance to antibiotics, the need to design effective treatments for *C. difficile* is urgently highlighted. One of the alternative approaches to antibiotic treatment is antibody-mediated therapy, including bezlotoxumab (the first and only FDA-approved human monoclonal antibody (mAb) to prevent rCDI) (17), mAb cocktails (18, 19), polyclonal antibodies (IGIV: immune globulin intravenous) (1, 20, 21), and active vaccination (22, 23) that can be effective for the treatment or prevention of CDI (24). Antibody therapy for CDI has many advantages over using conventional antibiotics, the most important of which is maintaining the intestinal microbial balance, composition and diversity (**Figure 1D**) (25). For instance, metronidazole is generally active on anaerobic bacteria like *Bacteroides* and *Bifidobacterium* (26), and vancomycin have also shown antimicrobial activity against *Bacteroides* and *Enterococcus* spp., also fidaxomicin, despite it is known as a specific anti-*C. difficile* agent, possesses antibacterial activity against *Bacillus* spp., *Bifidobacterium*, *Enterococcus* spp., and *Lactobacilli* (28). Accordingly, antibiotic-mediated gut dysbiosis can facilitate the germination of *C. difficile* spores in the patient’s gut, which may consequently lead to induction of rCDI cycle (29, 30). Moreover, the end of each course of traditional antibiotic therapy for CDI is regarded as the main period of patients’ vulnerability to CDI relapse (31). Notably, even if the over-mentioned disadvantages related to antibiotic therapy are ignored, treatment with antibiotics are not sufficiently effective in the treatment of rCDI and also the prevention of *C. difficile* spread (32, 33). Thus, development of efficient antibody-based therapies can allow to preserve the microbiota integrity and also reducing CDI recurrences. In recent decades, antibodies have been widely used as tools for basic research (34), diagnosis (35–40), and therapy (41–45). At present, the recombinant antibodies (rAbs) technology helps to develop more cost-effective antibodies (46–48). Here, we comprehensively discuss the latest research contributed to rAbs technology and the precise role of rAbs in the antibody-mediated therapy for CDI.

## Recombinant antibody technologies

**Figure 2:**
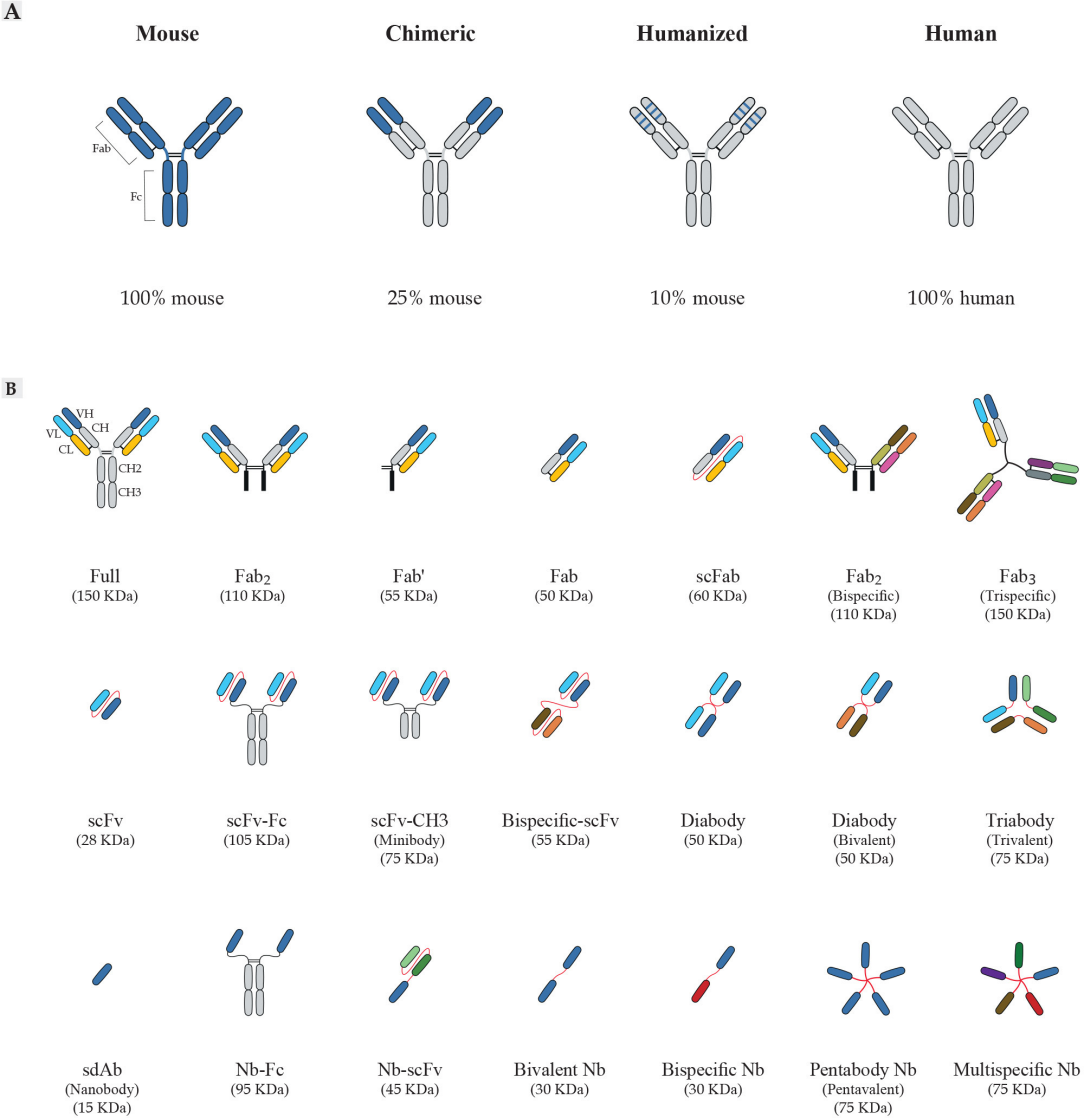
Schematic overview of antibody formats. **(A)** Antibody humanization from murine antibodies (blue domains) to fully human antibodies (gray domains). Chimeric antibodies are formed by fusing sequences of murine variable domain **(V)** regions to human constant **(C)** regions. Humanized antibodies are generated by grafting sequences of murine CDR to the human V-framework regions and expressed with human C regions. **(B)** Representation of recombinant antibody fragments. The design of small recombinant antibodies based on the VH and VL domains of the parental mAbs is provided by antibody engineering. Here, the basic types of antibody fragments including Fab, scFv, and sdAb, and their derivatives are represented. Fabs are composed of VL and a constant CL linked to VH and CH1 by a disulfide bond between the CL and CH1 domains. There are other formats based on Fabs, including Fab´ composed of a structure similar to Fab; scFabs composed of VL and CL linked to VH and CH1 by a flexible glycine-serine linker (Gly4Ser)3; bispecific Fabs fragments composed of 2 × Fab fragments joined by a disulfide bond; trispecific Fab fragments composed of 3 × Fab fragments joined by a disulfide bond with the paratope specificity for more than one antigens. scFvs are composed of VL linked to VH by a flexible glycine-serine linker (Gly4Ser)3. scFvs can be engineered to generate multivalent or multi-domain structures, including scFv-Fc composed of scFv to the Fc region of the antibody; scFv-CH3 composed of scFv to the CH3 region of the antibody; diabodies composed of 2 × scFv fragments joined by the shortening of the linker peptide; bispecific-scFv composed of 2 × scFv fragments joined by a flexible glycine-serine linker (Gly4Ser)3; forms of multimeric scFv composed of multiple scFv fragments with the paratope specificity for more than one antigens. sdAbs or nanobodies (Nb) composed of VH and devoid of the VL chain completely. The examples of other sdAb formats engineered are Nb-Fc composed of sdAb to the Fc region of the antibody; Nb-scFv composed of sdAb to scFv fragment of the antibody; bivalent Nb composed of 2 × Nb fragments; bispecific Nb composed of 2 × Nb fragments with the different paratope specificity; multispecific Nb composed of multiple Nb fragments with the different paratope specificity for more than one antigens. mAbs, monoclonal antibody; CDRs, complementarity-determining regions; CH, constant domain of the heavy chain; CL, constant domain of the light chain; VH, variable domain of the heavy chain; VL, variable domain of the light chain Fc, fragment crystallizable region; Fab, fragment of antigen-binding; scFv, single-chain fragment variable; sdAb, single-domain antibody; Nb, nanobody.

The typical antibody molecule contains three functional components, two fragment antigen binding domains (Fabs) and the fragment crystallizable (Fc) (**Figure 2**) (49). The antigen recognition process is accomplished by the six complementarity determining regions (CDRs) that make up antigen-binding region (paratope), and are located at the tips of the Fabs, highlighted in the variable regions of heavy and light domains (V_H_ and V_L_, respectively); namely, CDRH1, CDRH2, CDRH3, CDRL1, CDRL2, and CDRL3 (50). An antibody-antigen interaction is formed by foundation of non-covalent forces between the paratope of antibody and the antigenic determinant (epitope) of antigen, which also determine the affinity of antibodies. Notably, the length and composition of the CDR sequences are highly variable, especially in the CDR3 (51–54).

Since the last decades, polyclonal antibodies (pAbs) and mAbs have been used for diagnostic and therapeutic purposes, so-called theranostics applications (55–58). The function of antibodies is based on their high affinity and specific binding to target molecules, which make them reliable therapeutic/diagnostic tools (57, 59). In this regard, the hybridoma technology, known as mAb production technique, has been widely used for the production of antibodies with desired characteristics (60). Although the hybridoma technology has been applied successfully in numerous cases, it has many shortcomings. Most antibodies provided by hybridoma are murine antibodies (100% mouse protein), limiting their therapeutic applications due to human anti-mouse antibody reaction (HAMA) (61). However, this problem can be modified by manipulating the sequence and structure of the recombinant antibody technologies for production of chimeric antibodies, humanized antibodies, use of transgenic animals (i.e. XenoMouse technology), and surface display technologies (62–64). These methods are briefly described below.

### Chimeric, humanized, and fully human antibodies derived from mice

Cloning of immunoglobulin (Ig) gene segments was first performed in 1977 (65) and after that, in 1984, chimeric antibodies were produced by fusing the antigen-binding variable domains of a mouse mAb to constant domains of human antibodies (Fc fragment), which is composed of approximately 75% human and 25% mouse protein (66, 67) as presented in **Figure 2A**. Some advantages of chimeric antibodies are as follows: they retain the specificity of the parental mouse antibodies, and they have very low immunogenic properties for antibody administration in clinical trials (59), and longer half-lives compared to the parental antibodies (68). Successful performance of these antibodies shown by many studies, has led to their widespread use in diagnostics or therapy for several diseases (59, 61).

The first report on humanized antibodies was published in 1986. Humanized antibodies were then constructed by grafting the gene segments of the CDRs of mouse-sequence origin into the human framework regions with highest similarity to the original mouse framework (**Figure 2A**). This results in an antibody with 90% human and 10% mouse amino acid sequence (69, 70). Although the specificity of these antibodies was similar to mAbs, their affinity was lower. This can at least be partially overcome by different methods like secondary directed mutagenesis after humanization (71). Humanized antibodies are used for treatment of various diseases including cancers, autoimmune disorders, and infectious diseases (61, 72–75).

Transgenic technology for antibody production was first suggested by Alt et al. (1985), while Green et al. (1994) for the first time reported the generation of mice with germline modifications (76, 77). In transgenic mice, human Ig loci is introduced into the mouse genome instead of the respective murine counterpart (78), and immunization of mice with a specific antigen leads to activation of B lymphocytes producing human antibodies. The mAb isolation approach in this technique is similar to hybridoma, except that the entirely human antibody is produced in established cell lines. Production of completely humanized antibodies has led to widespread use of this method for therapeutic purposes (59, 79).

### Recombinant antibody fragments

Other strategies that overcome the disadvantages of hybridoma, e.g. being time-consuming and inefficient to generate antibodies toward toxic antigens, are techniques developed based on *in vitro* antibody generation. These techniques, known as surface display technologies, were invented concomitant to the application of humanized antibodies. The generation of antibodies by display technologies (e.g. mammalian cell display, ribosome display, yeast display, and phage display) is based on the interaction of displayed antibody fragments with an antigen *in vitro.* For this approach diverse antibody gene libraries are used (48, 80). Typically, the recombinant antibody libraries are constructed from different sources, including B cell repertoire derived from an immunized donor (immune libraries) (81, 82), pre-immune repertoires (naive libraries) (81, 83, 84), and synthetic design (synthetic libraries) (85, 86). Immune libraries are usually constructed to select a specific antibody against a target antigen in medical research (87) and exhibit more advantages than naive display libraries, including the increased likelihood of selecting antibodies with high affinity/stability, and *in vivo* affinity maturation (88).

An advantage of display technologies is its ability to achieve specific antibodies, which can be improved along with recombinant DNA technology and antibody engineering through cloning and expression of a variety of antibody fragments in different expression systems such as bacteria, yeast, and mammalian cells (48, 89–91). Furthermore, complementary DNA (cDNA) encoding antibodies are stable in this method and can be stored for several years (49). In display technologies, the size of the antibody molecule is reduced and only the intact antigen-binding site (paratope) is preserved, such as fragment antigen-binding (Fab), single-chain fragment variable (scFv), and single-domain antibodies (sdAb) (46, 49) as presented in **Figure 2B**. These antibody fragments can eventually improve the antibody’s therapeutic properties in terms of better penetration in tumor tissue, faster blood clearance for imaging purposes, lower retention times in non-target tissue, and inferior immunogenicity. The affinity and specificity of rAbs are similar to conventional antibodies, thus no avidity effect exists (49, 92). In addition, *in vitro* expression systems for production of antibody fragments can help to achieve sufficient amounts of antibodies for diagnostic and therapeutic purposes (49, 93, 94). Since these technologies depend on *in vitro* screening rounds, they do not involve the immune system (94). Another point is that application of rAb system allows to construct new recombinant proteins such as bivalent antibodies (95, 96), multivalent antibodies (97–99), and bispecific antibodies (100), for different purposes.

A unique feature of rAbs is their high affinity and specificity for target molecules. This feature has led to their successful use in therapeutic grounds, so that different antibody fragments have been generated by rAb techniques and successfully applied in different fields (93, 94, 101). There are more than 500 antibodies under clinical investigations worldwide for various diseases like autoimmunity and inflammation, cancer, organ transplantation, infectious diseases, diabetes, arthritis, and hypercholesterolemia, and as antidote against several toxins (61, 102–104).

The popular types of antibodies produced by display technologies are Fab, scFv, and sdAb (92). In the past two decades, scFv technology has been widely used for targeting haptens (105, 106), proteins (107, 108), carbohydrates (109, 110), receptors (111, 112), tumor antigens (113), and viruses (114), and they showed good potential in therapeutic and diagnostic applications. Currently, based on Therapeutic Antibody Database (http://tabs.craic.com/) more than 100 scFv antibodies are in human clinical trials, a few of which, blinatumomab (anti-CD19, CD3), brolucizumab (anti-VEGF-A), avelumab (anti-hPD-L1), belimumab (anti- BAFF, BLyS), darleukin (anti-L19-IL2), tralokinumab (anti-IL-13), briakinumab (anti-IL-12, IL-23), and atezolizumab (anti-PD-L1) are approved by FDA.

In parallel, successful application of Fabs has been shown against different diseases; for example, ranibizumab is a humanized mAb Fab derived from phage display library and it was approved in 2006 for inhibiting vascular endothelial growth factor A (VEGF-A) (115).

Recently, sdAb, named VHH or nanobody, have received considerable attention and often are produced by constructing antibody libraries from immunized animals such as camel, llama, or alpaca (116). The major advantage of sdAbs is their high capacity to penetrate specific target cells, e.g., solid tumors, diseased or infected cells (117). Importantly, sdAbs can easily be constructed as bispecific or multispecific formats which have multiple functions (116, 118–120); however, the main disadvantage of these formats of antibodies is their short half-life and rapid elimination from circulation (121). Furthermore, the sdAbs have been applied as high potential tools for different purposes, including treatment of infectious and inflammatory diseases (120, 122, 123), and detection of toxins such as cholera toxin (CT) (124).

#### Surface display platforms

As mentioned above, different surface platforms, e.g., mammalian cell display, ribosome display, yeast display, and phage display, are used for screening recombinant antibody libraries and each has different advantages and disadvantages (48, 89). The common method used in different platforms is the same for displaying the libraries and contains several rounds of screening for selecting specific antibodies (48). The main differences in platforms are related to the differences in the diversity of libraries, different efficiencies in screening, and the possibility of antibody maturation (48). In particular, yeast and mammalian displays can increase the affinity maturation of antibodies due to the ability of post-translational modifications in displayed fragments (48, 89, 125). Moreover, the main advantage of these platforms is their compatibility with fluorescence-activated cell sorting (FACS), leading to precise control over screening and selecting high-affinity antibodies, which bind specifically to the targeted antigen (125, 126). However, multivalent binding has been reported as a significant drawback of yeast or mammalian cell platforms because of the presence of antibody density on their cell surfaces. This disadvantage allows for isolating antibodies with lower affinity compared to monovalent techniques, e.g., phage display (48). Additionally, the construction of large libraries is challenging in these platforms, as far as the number of unique antibody copies in the yeast or mammalian libraries is several orders of magnitude lower than in phage libraries, leading to the selection of few candidates in the panning process (89, 127).

The phage, ribosome, and bacterial display libraries have been constructed with more than 10^11^ clones, allowing for the generation and screening of a huge diversity of clones (48, 86, 128). Antibody expression in these systems, especially in phage display, is considered a straightforward process with many advantages (95, 96). Moreover, since gene manipulation in these platforms is easy, the generation of antibodies with higher affinity and specificity is more feasible than other recombinant technologies (49, 129). Notably, over the past decades, phage display has been the most widely used system for the production of rAbs and was awarded 2018 Nobel Prize in Chemistry. Application of phage antibody libraries has been reported for identification of various targets, including haptens, and foreign and self‐antigens (106, 130, 131). Moreover, the use of phage display for therapeutic purposes has received great attention. The first FDA-approved therapeutic antibody produced based on this technique was adalimumab (Humira), that was approved as an anti-tumor necrosis factor α (TNFα) human antibody against rheumatoid arthritis (RA) in the US in 2002 (86, 132). Overall, 15 therapeutic antibodies isolated from phage display libraries have been approved by the US FDA (**Table 1**), and over 110 antibodies are currently in clinical evaluation stage (http://tabs.craic.com/).

**Table 1:** FDA-approved human mAbs derived from phage display libraries.

Product name	Trade name	Fragment antibody	Final antibody format	Target	Marketing company	Application	Approval	Reference
Adalimumab (D2E7)	Humira	scFv	IgG1-κ	TNFα	AbbVie	Rheumatoid arthritis	2002	(133)
Ranibizumab	Lucentis	Fab	Fab	VEGFA	Roche/ Genentech	Macular degeneration growth factor A	2006	(134)
Belimumab	Benlysta	scFv	IgG1-λ	BLyS	GSK	Systemic lupus erythematous	2011	(135)
Raxibacumab	Abthrax	scFv	IgG1-λ	Anthrax PA	GSK/HGSI	Inhalation anthrax	2012	(44)
Ramucirumab	Cyramza	Fab	IgG1-κ	VEGFR2	Eli Lilly	Gastric cancer, colorectal cancer and non-small cell lung cancer	2014	(136)
Necitumumab	Portrazza	Fab	IgG1-κ	EGFR	Lilly/ AstraZeneca	Squamous non-small cell lung cancer	2015	(137)
Atezolizumab	Tecentriq	–	IgG1-κ	PD-L1	Roche	Metastatic lung cancer, Renal cancer	2016	(138)
Ixekizumab	Taltz	Fab	IgG4-κ	IL-17A	Eli Lilly	Psoriasis	2016	(139)
Guselkumab	Tremfya	Fab	IgG1-λ	IL-23, subunit p19	Janssen Biotech	Plaque psoriasis	2017	(140)
Avelumab	Bavencio	Naive Fab	IgG1-λ	PD-L1	Serono/Pfizer	Merkel cell carcinoma, metastatic urothelial carcinoma	2017	(141)
Lanadelumab	Takhzyro	Fab	IgG1-κ	pKaI	Dyax Corp.	Hereditary angioedema attacks	2018	(142)
Emapalumab	Gamifant	scFv	IgG1-λ	IFNγ	NovImmune SA	Primary hemophagocytic lymphohistiocytosis	2018	(143)
Moxetumomab pasudodox	Lumoxiti	scFv	Murine IgG1 dsFv	CD22	MedImmune/AstraZeneca	Hairy cell leukemia,	2018	(144)
Caplacizumab	Cablivi	Nanobody	Humanized V_H_-V_H_	VWF A1 domain	Sanofi/Ablynx	Acquired thrombotic thrombocytopenic purpure	2018	(145)
Tralokinumab	Adtralza	scFv	IgG4-λ	IL-13	AstraZena	Atopic dermatitis	2021	(146)

TNF-α, tumor necrosis factor-alpha; VEGFA, vascular endothelial growth factors A; BLyS, B-lymphocyte stimulator; PA, protective antigen; VEGFR2, vascular endothelial growth factor receptor 2; EGFR, epidermal growth factor receptor; PD-L1, programmed death-ligand 1; IL-17A, interleukin 17A, IL-23, interleukin-23; pKaI, plasma kallikrein; IFN-γ, interferon gamma; CD22, cluster of differentiation-22; vWF-A1, A1 domain of von Willebrand Factor (vWF).

Although phage display has many advantages, the main limitation of this method, which is also seen in the ribosome and bacterial display techniques, is that antibody interrogation is based only on the binding properties of binders and not on functional features (147–149). Moreover, until recently, these technologies were unable to preserve the cognate V_H_–V_L_ pairing, because obtaining paired information requires determining the antibody sequence at individual cell level (150, 151). This limitation sometimes leads to rising antibodies with inferior selectivity and weaker biophysical features compared with human antibodies or Igs (152), however, this disadvantage can be adjusted in immune libraries due to the pre-enrichment and *in vivo* affinity maturation (88).

Over the last decade, single-cell technologies combined with next-generation sequencing (NGS) approaches have been introduced as a high-throughput screening of antibodies, which are highly boosted by microfluidic platforms (153, 154). Surface display platforms combined with single-cell technologies can provide open-source computational tools to interrogate more in-depth V_H_–V_L_ sequences in display libraries (150, 155) and allow screening of diverse antibodies based on their functional properties, affinity, and biophysical characteristics (151). In the following, single-cell RNA sequencing (scRNA-seq) technologies are discussed briefly.

#### Single-cell RNA sequencing technologies (scRNA-seq)

**Figure 3:**
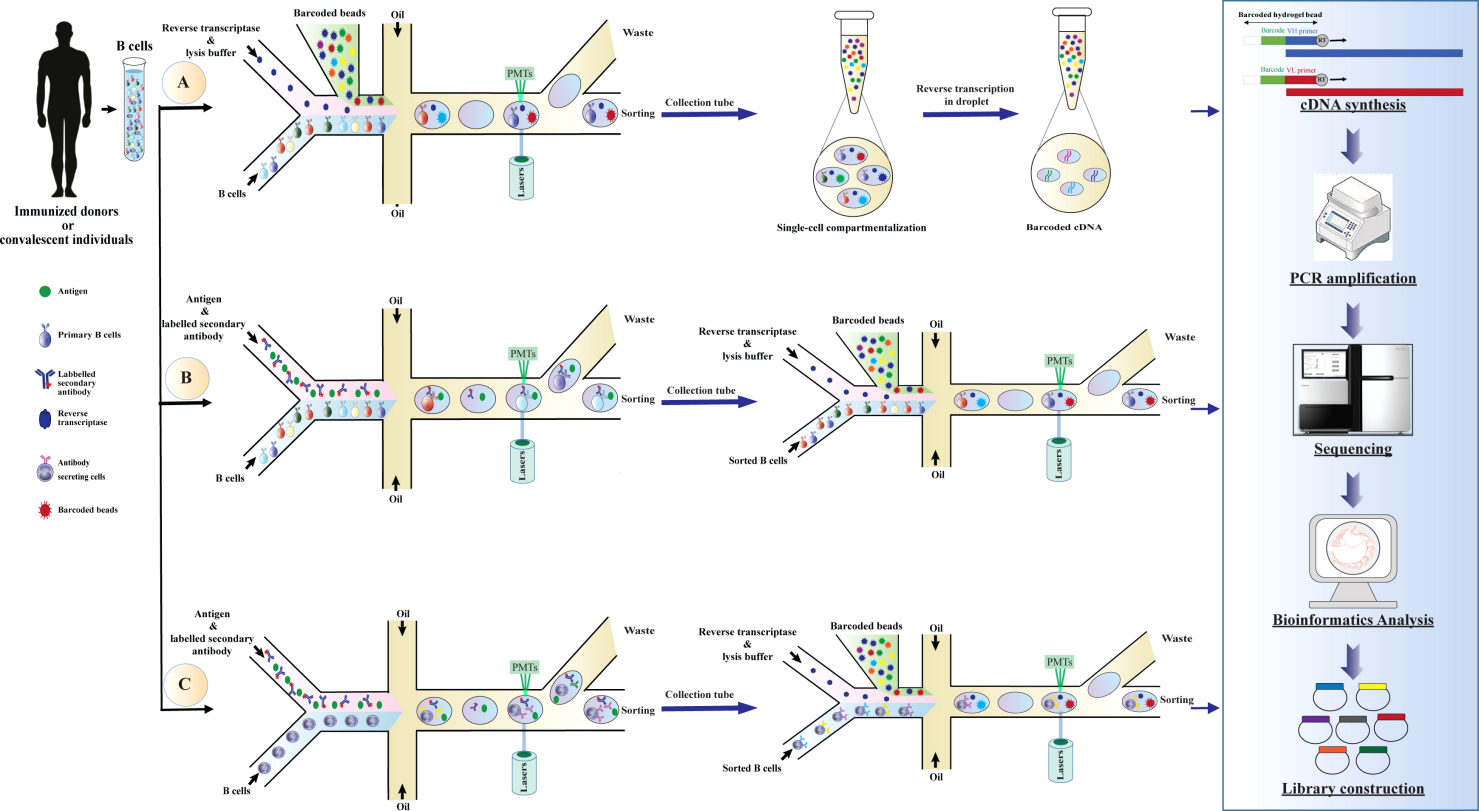
Schematic overview of application droplet-based microfluidics for high-throughput screening of human B cells repertoire and library construction. Development of antibody libraries is based on screening **(A)** human B cells repertoire, **(B)** B cells binding to a specific antigen, or **(C)** B cells secreting antibodies binding to a specific antigen. The basic steps of the method are as follows: droplets with a specific fluorescence intensity are deflected into the collection channel through electric fields. The selected cells are re-compartmentalized in the droplets simultaneously with a hydrogel bead coupled with uniquely barcoded polyT primers to generate droplets containing a single cell and a single bead. After cell lysis, reverse transcription is conducted for the V_H_ and V_L_ domains in the droplets. Since the cDNAs synthesized from each cell are conjugated with a unique barcode (corresponding to mRNAs produced in the droplets), cognate V_H_ and V_L_ pairs can be identified by NGS and subsequently used for library generation. cDNA, complementary DNA; mRNA, messenger RNA; V_H_, variable domain of the heavy chain; V_L_, variable domain of the light chain, NGS, next-generation sequencing; PMTs, photomultiplier tubes.

Over the last decade, the application of NGS approaches to explore antibody repertoire has been extensively investigated, some of these approaches can recover the cognate V_H_-V_L_ pairing and identify the genotype of antibodies (150, 152, 156–158). However, there is a need for a system that can support high-throughput phenotype-genotype screening (159, 160). Recently, single-cell platforms have been applied to probe the diversity of V_H_-V_L_ chains of antibodies and to assess the specificity of antibodies at large scales (150, 152, 153, 157). These technologies can maintain the genotype and phenotype linkage of antibodies and allow them to be compatible with high-throughput RNA sequencing output (155). The basic steps for scRNA-seq include sample collection, single-cell isolation and capture, cell lysis, barcoded reverse transcription, cDNA amplification sequencing, bioinformatics analysis, and library preparation (**Figure 3**). The most widely used techniques for single-cell isolation and capture include FACS, microfluidic system, and magnetic-activated cell sorting (154).

Droplet-based microfluidics is one of the most efficient technologies for single-cell studies and has shown great potential for functional high-throughput screening, genomics studies, and comprehensive analysis of the immune repertoire diversity (147, 159). These technologies have conceptual advantages, such as portability of equipment, consuming just a few cell numbers for performing large-scale studies, high separation efficiency, high degree of automation, fast analysis, and enabling the preservation of phenotype-genotype linkage within the droplet (159, 161). These advantages make droplet microfluidics a preferable tool for biomedical research and single-cell studies (150, 155). In these systems, B cells are encapsulated into droplets by a co-flow emulsion droplet microfluidic chip for mRNA capturing, and then heavy and light chains of the antibody in a single cell are characterized by a hydrogel bead coupled with uniquely barcoded primers (154, 159). Subsequently, the barcoded cDNAs are sequenced and exploited for generating recombinant antibody libraries, e.g., phage display and yeast display libraries (**Figure 3**) (150, 152, 162).

In this way, microfluidic technology can be applied for library construction from B cell repertoire derived from immunized donors or convalescent individuals, supporting native chain-paired library generation and direct screening of antibodies (**Figure 3A**). This method can easily check millions of primary human B cells derived from the native repertoire into an automated and sensitive screening platform (150, 152). Recently, applications of this technology for constructing several libraries have been reported, including coupling recombinant repertoires obtained from the microfluidic platform with phage display technology to rapidly screen specific antibodies for various targets such as influenza hemagglutinin (150) and programmed cell death protein 1 (PD-1) (162). Also, droplet-based microfluidics combined with yeast display technology are exploited for selecting neutralizing antibodies for human immunodeficiency virus type 1 (HIV-1), Ebola virus glycoprotein, and influenza hemagglutinin (152). This method can be applied as a powerful tool for screening high-affinity antibodies for therapeutic approaches (147, 163, 164).

Additionally, droplet-based microfluidics can be used for high-throughput single-cell screening for the selectivity of antibodies unique to each B cell in the presence of the target antigen. This system can be applied for the screening of primary B cells or Ig-secreting cells such as plasma cells obtained from immunized donors (28) or plasmablasts from human peripheral blood (159, 165, 166). In these systems, B cells have been co-cultivated with antigen-expressing cells or antigen-coated beads in the presence of fluorescently labeled secondary antibodies. B cells or antibodies secreted from them can bind to the antigen and generate a specific fluorescence intensity. Specific B cells can be identified based on intensity fluorescence peak and used for constructing specific libraries (154) as presented in **Figures 3B, C**. The successful development of these technologies has been reported in several studies (157, 159, 160). This technology facilitates the selection of high-affinity antibodies with distinct functional features for therapeutic purposes and can help construct libraries with high specificity and functional activities (154, 162). Taken together, single cell technology based on microfluidic systems can be integrated with display techniques or applied to generate libraries containing matured and specific antibodies with high diversity and affinity.

## Development of recombinant therapeutic antibodies for *C. difficile*

Previous studies on the pathogenicity of *C. difficile* have confirmed that its major virulence factors are the large secreted glucosyltransferase protein toxins TcdA and TcdB, which are encoded within a 19.6-kb pathogenicity locus (PaLoc) (167, 168). TcdA and TcdB are involved in the development of inflammatory response associated with the production of chemokines and cytokines, neutrophil influx, fluid secretion, and cell damage and death (6, 169). In addition to the above-mentioned toxins, some more virulent strains produce a binary toxin or *C. difficile* transferase (CDT), which is an actin-specific ADP-ribosyltransferase (170). Other factors including cell wall proteins (Cwps that mainly act as adhesions), flagellin (FliC), flagellin filament cap protein (FliD), and S-layer proteins (SLP) are also involved in the pathogenicity of *C. difficile* that contribute to colon localization, and evasion of the immune system surveillance (171). These factors may play a role in the initiation of bacterial pathogenesis trough inducing inflammatory responses and interactions with toll-like receptors (TLRs) (169). Furthermore, several extracellular enzymes that are produced by *C. difficile* may be important in normal physiological processes and survival of the bacterium in the GI tract, however, their decisive role in pathogenesis is still uncharacterized. Noteworthy, typical clinical symptoms of CDI can only be caused by strains producing TcdB, or both TcdB and TcdA (172). Moreover, in many studies, it is proven that the major virulence factor in CDI is TcdB, which can alone induce severe organ damage *in vivo* (6, 168, 173), but it should be noted that the virulence features are also retained in strains expressing only TcdA (12, 174, 175). Nevertheless, the abundance of TcdA^–^B^+^ strains isolated from patients is higher than TcdA^+^B^–^ strains (176, 177). According to this, considerable efforts have been made to use antibodies that can directly target toxins instead of the bacterium, thus, both TcdA and TcdB can be used as promising target antigens in antibody production (178–180).

### Antitoxin A/B antibodies

Over the past 30 years, several antibodies have been developed against TcdA or TcdB, and their effectiveness has been evaluated in animal models, and some of them have shown therapeutic value in CDI treatment. A summary of the antibodies used in the study of *C. difficile* infection is presented in **Table 2**. Among these antibodies, the C-terminal domain of both TcdA and TcdB, i.e., building combined repetitive oligopeptide structures (CROPs), which is involved in the binding of toxins to carbohydrate receptors on the surface of host cells, have been mostly used as target antigens for antibody production (12). Based on several previous studies, immunization of animal models using the CROPs of *C. difficile* toxins could induce production of toxin neutralizing antibodies among challenged animals with either toxins or live bacteria (222–225). The application of different antibodies, e.g. pAbs, mAbs, and rAbs, has been widely investigated in CDI treatment. Due to the effectiveness of serum therapy in the treatment of several bacterial diseases (226), this type of therapy was also suggested for CDI. Several studies have investigated the therapeutic efficacy of pAbs derived from animals that were immunized with mutants of TcdA and TcdB (181, 227), and the results showed the effectiveness of this method in reduction of CDI incidence in infected animals. Furthermore, the use of serum antibodies against TcdA and TcdB was suggested for patients with CDI (20, 228). In this regard, the application of a polyclonal ovine antibody that binds to toxins and neutralizes their effects has been successfully applied for CDI treatment. Interestingly, this method did not exhibit an immunogenic effect on the patients (189). Moreover, serum antibodies derived from healthy blood donors have been investigated in many studies (229–231), some of these studies demonstrated that this method can be successfully used as an oral treatment in patients with severe CDI who were refractory to standard treatments (232). Recently, bovine antibodies from hyperimmune colostral milk can be regarded as a powerful orally-administered drug candidate that is currently in the clinical development (190, 227). However, efforts to produce more effective therapeutic antibodies are continued since antibodies must have a clear advantage over other available treatments to be accepted as a promising agent by clinicians and regulatory authorities.

**Table:** Table 2 Summaries of antibodies used in the study of *Clostridioides difficile* infection.

Antibody type	Antibody name	Target	Antibody source	Neutralizing *in vitro*	Protective *in vivo*			Summary	Reference(s)
					Animal model	Ab administration route	Protection		
**Polyclonal antibodies**	Bovine IgG	Culture filtrate	Cow colostrum	N/d	Hamster	Oral	Yes	Hyperimmune IgG fraction from bovine colostrum protected hamsters with high efficacy.	(181)
	Bovine IgG	Inactivated TcdA, culture filtrate	Cow colostrum	Yes	Rat	Orogastric dosing	Yes	Bovine colostrum IgG neutralized the effects of TcdA or culture filtrate (TcdA and TcdB).	(182)
	IgY	rTcdA/rTcdB	Chicken	No	Hamster	Oral	Yes	Antibodies that bind to the C- terminal domain of toxins were the most effective. Anti-TcdA alone was able to protect ~70% hamsters, but the antibody combination of anti-TcdA and anti-TcdB protected ~100% hamsters.	(183)
	Anti-TcdB	rTcdA/rTcdB	Mouse	N/d	Hamster	I.P.	Yes	Serum antitoxin antibodies mediate systemic and mucosal protection from *C. difficil*e disease in hamsters.	(184)
	Bovine colostrum	culture filtrate	Cow colostrum	N/d	Hamster	Oral	Yes	Orally Administration colostrum IgG for 3 days conferred ~90% protection to hamsters. Recurrent CDI were not observed in any patients.	(185)
	IgG	SLP	Rabbit	Yes	Hamster	Orogastric dosing	Yes	Anti-SLP antibodies increased survival compared with control groups and modulated the course of CDI.	(186)
	IVIG	–	Human immunoglobulin	N/d	Human	Oral	Yes	Human immunoglobulin protected patients. It was highly effective in treating patients with multiple recurrences of CDI.	(187)
	IgY	FliC, FliD, Cwp84	Chicken	Yes	Hamster	Oral	Yes	FliD-specific IgY significantly protected hamsters from CDI.	(188)
	IgG	TcdA/TcdB	Sheep	Yes	Hamster	I.P.	Yes	Administration of anti-TcdA sera reduced the symptom severity but conferred no protection to hamsters against death. The combination of anti-TcdA and anti-TcdB protected ~50 to 90% hamsters after administration of 75 mg of each antibodies.	(189)
	HBC	spores, vegetative cells, rTcdB	Cow colostrum	Yes	Mouse	Oral	Yes	Administration of TcdB-specific colostrum prevented CDI in Mouse. Coadministration TcdB-specific colostrum with spore or vegetative cell-targeted colostrum reduced recurrence rate up 67%.	(190)
	Bovine colostrum	TcdA/TcdB	Cow colostrum	N/d	Hamster	Oral	Yes	Bovine colostrum protected ~50 to 100% hamsters. In addition to efficacy to treat primary CDI, it prevented of recurrent CDI in infected hamsters.	(191)
	OraCAb	rTcdA/rTcdB	Sheep	Yes	Hamster	Oral	Yes	The OraCAb neutralized toxin production in *in vivo* and *in vitro*. Coadministration of OraCAb together with vancomycin prevented simulated CDI recurrence in hamsters.	(192)
	Secretory IgA (sIgA)	TcdA/TcdB	Human	N/d	Hamster	Oral	Yes	Coadministration of human secretory IgA (sIgA) and vancomycin enhanced survival in hamsters challenged with *C. difficile.*	(193)
**Monoclonal antibodies**	PCG-4 IgG	TcdA	Mouse	No	Hamster	Oral	Yes	PCG-4 neutralized the effects of TcdA in *in vivo*. It binds to 2 epitopes of C-terminus of TcdA and blocks the binding of TcdA to Caco-2 cells.	(194) (195)
	G-2 IgG	TcdA/TcdB	Mouse	No	Hamster	Oral	No	G-2 binds to a shared epitope on TcdA and TcdB, but it did not neutralize either toxin.	(196) (194) (197)
	37B5 IgG	TcdA	Mouse	No	Rabbit Mouse	I.P.	No	37B5 neutralized the effects of TcdA in a rabbit ligated ileal loop assay, however, it did not protect mouse challenged with TcdA.	(198)
	A9, 141-2, C11	TcdA	Mouse	N/d	Mouse	I.V.	Yes	Antibodies Recognized C-teminal domin of TcdB. I.V. administration of antibodies protected Mouse from the effects of *C. difficile*.	(199)
	3358, 3359	TcdA (CROPs)	Mouse	N/d	Hamster	I.P.	No	Modestly neutralizing mAbs observed. The use of mAbs cocktail showed a better effect in neutralizing the toxin. Overall, there are no protection observed.	(200)
	A1H3	TcdA	Mouse	N/d	Piglet	–	N/d	A1H3 enhanced cell-surface recruitment of TcdA.	(201) (202)
	1G3, 1B5, 2D4, 2C7, 4A4, 5D8	TcdA (CROPs)	Mouse	Yes	Mouse	I.P.	Yes	A mixture of 4A4 and 5D8 was useful for detection of and protection against TcdA. 4A4 protected. 50% mouse challenged with purified TcdA, while in combination with 2C7 increased up ~90% protection. There is no protection by 5D8.	(203)
**XenoMouse** **antibodies**	Actoxumab (CDA1)	TcdA (CROPs)	Transgenic mouse (human IgG1)	Yes	Mouse Hamster Piglet Human	I.P.	Yes	A mixture of two actoxumab and bezlotoxumab was able to protect animal models (mouse, hamster, piglet, and human). No/poor efficacy observed to protect piglet/human when actoxumab used alone.	(18, 204–207)
	Bezlotoxumab (MK6072, CDB1, MDX-1388, 124-152)	TcdB (CROPs)	Transgenic mouse (human IgG1)	Yes	Mouse Hamster Piglet Human	I.P.	Yes	A mixture of two bezlotoxumab and actoxumab was able to protect animal models (mouse, hamster, piglet, and human). bezlotoxumab alone was capable of efficacious to reduce recurrence CDI rate in human.	(18, 204, 206, 207)
	A2, B1, B2	rTcdA/rTcdB	Transgenic mouse (human IgG1)	Yes	Hamster	I.P.	Yes	A mixture of A2 (anti-TcdA) with B1 and B2 (anti-TcdB) neutralized TcdA and TcdB in *in vitro* assays. 3-Mabs cocktail antibodies reduced mortality rate and disease severity in hamsters challenged with *C. difficile.*	(208)
**Humanized** **antibodies**	PA-50, PA-41	TcdA/Tcd, GTD	Mouse	Yes	Hamster	I.P.	Yes	Both antibodies neutralized of TcdA and B from multiple C. difficile ribotypes. A mixture of two PA-50(anti-TcdA) and PA-41 (anti-TcdB) protected~90 to 100% hamsters until day 39 postinfection.	(202, 209, 210)
	CA997	TcdA (CROPs)	mAb (humanized IgG1)	Yes	Hamster	I.P.	Yes.	CA997 neutralized TcdA from multiple *C. difficile* ribotypes. The combination CA997 with CA1125 and CA1151 protected hamsters with more potency than actoxumab/bezlotoxumab combination.	(19)
	CA1125,CA1151	TcdB (CROPs)	mAb (humanized IgG1)	Yes	Hamster	I.P.	Yes	A mixture of both mAbs neutralized TcdB. In combination with CA997 had Greater protection than actoxumab and bezlotoxumab combination.	(19)
	CANmAbA4, CANmAbB4, CANmAbB1	rTcdA/rTcdB	Mouse	Yes	Hamster	I.P.	Yes	The humanized anti-TcdA (CANmAbA4) and anti-TcdB (CANmAbB4 and CANmAbB1) antibodies neutralized both toxins in *in vitro*. Coadministration of CANmAbA4 and CANmAbB4 protected ~ 85% hamsters after administration of 50 mg/kg of each antibody.	(211)
**Recombinant antibodies**		TcdB	Human scFv	Yes	–	–	N/d	The scFv Fragment had high specificity for toxin B and no cross-react observed with non-toxigenic strains of *C. difficile*.	(212)
	A4.2, A5.1, A19.2, A20.1, A24.1, A26.8	TcdA (CROPs)	Llama VHH	Yes	–	–	N/d	VHH fragments neutralized TcdA. The mixture of VHH had more efficacy for toxin neutralization. Administration of *B. longum* transformed with A20.1 or A26.8 resulted in gut expression. There is No data for infection models.	(213–216)
	B5.2, B13.6, B15.5, B39	TcdB (CROPs)	Llama VHH	No	–	–	N/d	Anti-TcdB VHH were non-neutralizing *in vitro*. No data for infection models.	(213, 215)
	B4, B5, B12, B17	TcdB (CROPs)	Human V_L_	No	–	–	N/d	–	(213)
	ABA (AH3–E3–E3–AA6)	TcdA/TcdB	Alpaca VHH (bispecific, tetrameric)	Yes	Mouse	I.P.	Yes	Tetramer VHHs neutralized TcdA and TcdB in *in vitro*. Tetramer protected mice challenged with TcdA/B and *C. difficile.*	(217)
	B2, E2, G3, D8	TcdB: CROPS	Llama VHH	Yes	Hamster	Oral	Yes	VHHs (B2, G3, and D8) neutralized TcdB, while combinations of VHHs did not improve neutralizing potency. Administration of *Lactobacilli* displaying B2 and G3 resulted in gut expression. An administration model with *Lactobacilli* displaying B2 and G3 VHHs was protective.	(218)
	Anti-SLP	SLPs	Llama VHH	Yes	–	–	N/d	Anti-SPL fragments strongly bonded to different ribotypes of *C. difficile*.	(219)
	Anti-FLiC, Anti-FliD	FliC/FliD	Human scFv	Yes	–	–	N/d	scFv antibodies was strongly able to detect *C. difficile* 630 and also to inhibit bacterial motility.	(220)
	Anti-TcdB	TcdB	Human scFv, scFv-Fc	Yes	–	–	N/d	The epitopes of the neutralizing and non-neutralizing scFv fragments were identified and a new neutralizing epitope within the glucosyltransferase domain of TcdB was recognized.	(221)

*C. difficile*, *Clostridioides difficile*; mAb, monoclonal antibody; scFv, single-chain fragment variable; VHH, variable domain of heavy-chain antibody or nanobody; IgY, egg yolk antibodies; IVIG, intravenous immunoglobulin; HBC, hyperimmune bovine colostrum, SLP, surface layer proteins; FliC, flagellin Protein, FliD, flagellar capping protein; Cwp84, a surface-associated protein; LPS, bacterial lipopolysaccharides; CROPS, receptor-binding domain; GTD, glucosyltransferase domain; N/d, not determined; IP, intraperitoneal; IV, intravenous.

The earliest application of specific mAbs for *C. difficile* toxins in animal research was reported by Lyerly et al. (1986). This work showed that pre-mixing mAbs and oral administration of their admixture can completely protect hamsters against detrimental effects of toxin (194). So far, several antibodies have been generated against *C. difficile* and their effectiveness has been evaluated in various models of animals. Some of these studies proposed the use of anti-toxin antibodies as beneficial agents for *C. difficile* treatment, particularly for preventing the initial episodes of CDI and reducing the risk of rCDI (1, 182, 233). Most investigations on anti-toxins are based on antibody production from animal sources, while the clinical application of such antibodies requires humanization to reduce the potential immune-related adverse events (irAE) for human use. Presently, there is only one FDA-approved antibody to treat rCDI (bezlotoxumab), and the other antibodies are in the clinical or laboratory stages, which are discussed below.

### Bezlotoxumab: The first FDA-approved therapeutic monoclonal antibody for the prevention of CDI recurrence

The first specific human anti-toxin mAbs for TcdA and TcdB were reported by Babcock et al. in 2006, and today, they are known as actoxumab and bezlotoxumab, developed against TcdA and TcdB, respectively. In this work, human monoclonal IgGs (HuMAbs) were produced in transgenic mice immunized with inactivated TcdA, TcdB, and recombinant TcdB as antigens (204). Screening of various hybridomas *in vitro* and *in vivo* led to the isolation of specific antibodies against TcdA (CDA1) and TcdB (MDX-1388) that could significantly reduce hamster mortality in primary CDI treatment and CDI relapse models.

The evaluation of the safety and pharmacokinetic of HuMAb CDA1 confirmed that this antibody is safe at doses between 0.3 and 20 mg/kg. Interestingly, the combination therapy of HuMAb CDA1 and antibiotics could significantly decrease the hamster’s mortality (204). The high efficacy of these two antibodies in the protection of animals against CDI led to the initiation of the first human clinical trial for treating CDI by using CDA1 and MDX-1388, as far as, the placebo-controlled phase III trials, MODIFY I and II, were done to determine the efficacy of both antibodies in CDI patients (17, 234). After that, anti-TcdA (CDA1) and anti-TcdB (MDX-1388) were named actoxumab and bezlotoxumab, respectively. These clinical trials showed that actoxumab does not have clinical efficacy in clinical phase III (MODIFY II), while bezlotoxumab reduced CDI recurrence from ~40% in this phase (absolute rate reduction of ~10%) (17).

The safety and pharmacokinetic studies of bezlotoxumab confirmed the acceptable performance of this antibody. However, it should be noted that the assessment reports of the FDA and then the European Medicines Agency (EMA) demonstrated that administration of bezlotoxumab in subjects with baseline congestive heart failure, increased heart failure incidence and all-cause mortality compared to placebo-treated patients. Finally, in 2016, bezlotoxumab (Zinplava^©^) was approved by the FDA and EMA, for the prevention of rCDI in adult patients (≥18 years) (U.S. Food and Drug Administration, 2016 (https://www.fda.gov/). Notably, bezlotoxumab can only reduce the rate of CDI relapse to ∼40% compared to placebo and is unfavorable for treating acute CDI (Navalkele and Chopra, 2018). Therefore, bezlotoxumab can be applied as an effective therapy for preventing rCDI. However, the clinical effectiveness of the drug should be assessed in further studies (235).

Current international guidelines advocated the standard of care (SoC) antibiotics for CDI treatment, and metronidazole or vancomycin are recommended for mild to moderate CDI, and fidaxomicin for severe disease and/or multiple CDI episodes accordingly (13, 14, 27, 32, 236). Among the proposed antibiotics, fidaxomicin is the only specific antibiotic for *C. difficile*, however, there is no study to date that has compared the cost-effectiveness of fidaxomicin with bezlotoxumab. The only cost-effectiveness analysis is related to the comparison of the effect of fidaxomicin with standard therapy plus bezlotoxumab as reported by Lam et al. focusing only on rCDI (237). Additionally, it is proven that fidaxomicin plus bezlotoxumab has similar effect to other SOC antibiotics (i.e., vancomycin or metronidazole) plus bezlotoxumab (42, 238, 239). Notably, pharmacoeconomic analyses demonstrate that standard therapy plus bezlotoxumab could be cost-effective compared with standard therapy alone, especially in preventing of rCDI episodes in those >65 years of age, those with severe CDI, and immunocompromised patients (234, 237, 240–243). The results of a retrospective study on high-risk patients treated with bezlotoxumab have proved the clinical effectiveness of this antibody, in which 73% of the treated patients remained free of recurrence during a follow-up of three months (241). Additionally, bezlotoxumab administration to immunocompromised patients could prevent rCDI in 71% of these patients (243). Therefore, the adjunctive use of bezlotoxumab can be recommended in those patients with three or more risk factors promoting recurrence development.

### Toxin-neutralizing antibody that are in clinical phases

In addition to bezlotoxumab, some other antibodies are currently in clinical trial phases and some have been considered for oral use, thus, the number of such antibodies as promising therapies may increase in the future. For example, humanized murine toxin-specific mAbs reported by Marozsan et al. (2012), PA-50 (humanized anti-toxin A mAb), and PA-41(humanized antitoxin B mAb), were found as an acceptable choice for treating CDI and can be used as a non-antibiotic agent for improving CDI management (209). Additionally, in this work the authors showed that the application of a mixture of both PA-50/PA-41 in hamster models of CDI led to 95% long-term survival compared to 0% survival of animals treated with the standard antibiotic vancomycin. Also, the efficacy of the combination of PA-50/PA-41 was much higher than that of the combination of actoxumab and bezlotoxumab. Interestingly, the potency of PA-50 was significantly higher than actoxumab *in vitro*, because of its multivalent interactions with TcdA. PA-41 is also significantly more potent compared to bezlotoxumab. Evaluation of the efficiency of these mAbs against different ribotypes (RTs) of *C. difficile in vitro*, e.g., RT001, RT002, RT003, RT012, RT014, RT017, RT027, and RT078, revealed their broad neutralizing activity; such properties introduce these mAbs as attractive candidates in non-antibiotic therapy of CDI (209). As shown in this study, a combination of antibodies could increase the effectiveness of antibody therapy. The combination of antibodies directed against different target structures/epitopes/domains may have a synergistic effect, thus increasing the performance of antibody therapy (244). This hypothesis was examined and proven in other studies (19, 208, 211). Importantly, some clinical trials demonstrated that the combination of a cocktail of anti-toxins A and B antibodies and antibiotics like vancomycin, significantly decrease the recurrence rate compared to antibiotic therapy alone for CDI (1, 18, 234, 242). This indicates great potential of co-administration of anti-toxin antibodies and antibiotics over standard antibiotic therapy.

### Recombinant fragment antibodies neutralizing toxin A/B

In addition to humanized antitoxin and mouse mAbs under development and their combination application, rAb fragments can be used as alternative immunotherapeutic agents for treating CDI. In the previous part, we described the advantages of using rAb fragments. Today, rAb technology is known as a rapid and high-performance approach to introduce the next generation of immunotherapy agents. The rAb fragments can be applied as a suitable option to bind epitopes that are inaccessible for conventional antibodies due to their high tissue penetration capability (245, 246). Additionally, the small size of the rAb allows them to access immuno-silent cavities in enzymes and receptors (247). These properties of rAbs have made them favorable agents for *C. difficile* toxin neutralization, which may have even greater efficacy in the GI tract (213, 248).

As mentioned earlier, scFv antibodies have applied to neutralize potent toxins. However, there are few publications about the isolation of scFv antibodies that bind *C. difficile* toxins. Deng et al. (2003) reported the isolation of toxin B-neutralizing scFv from a scFv library by phage display technology; however, the work did not progress beyond binding assays (212). Recently, antibodies targeting different domains of TcdB were isolated (221). After conversion to the IgG-like bivalent scFv-hFc format 31 humans, anti-TcdB antibody fragments were further characterized for TcdB binding and neutralization. Analysis of the epitopes of these antibody fragments to identify neutralizing and non-neutralizing epitopes was done using domain mapping, TcdB fragment phage display, and peptide arrays. These results provided new insights into the function of different toxin regions and their relevance to neutralization and toxicity because a new epitope for toxin neutralization within the N-terminal glucosyltransferase domain (GTD) of TcdB was identified. The bivalent scFv-Fc formats have been constructed and characterized in several studies (96, 249), their results showed the conversion of antibody candidates into an IgG format like bivalent scFv-Fc is recommended for further validation and characterization of selected fragments. Moreover, it seems that scFv-Fc formats provide rapid screening of many candidate antibodies, thus it is preferable over full IgG format (49, 244, 250).

Similarly, the application of sdAbs for treating toxin-mediated diseases has been assessed by several groups (251, 252), and antitoxin sdAbs were also successfully generated to neutralize *C. difficile* toxins in recent studies (180, 213, 253). The first isolation of specific sdAb fragments for detecting *C. difficile* toxins was reported by Hussack et al. (2011 and 2012). In this work, specific llama VHHs against CROPs fragments from each toxin were isolated from an immune phage display library. The results showed that six of seven selected anti-TcdA sdAbs inhibited the cytotoxicity of TcdA when added at 1000 nm concentration, whereas none of the seven selected anti-TcdB sdAb fragments blocked TcdB cytotoxicity. Interestingly, the mixture of anti-TcdA sdAbs improved toxin neutralization at lower sdAb concentrations (213, 248). In another work, Murase et al. described the isolation of sdAbs binding to CROPs of TcdB (215). Antibody fragments isolated in this study demonstrated different binding properties, and some of them e.g. B5.2, B13.6, and B15.5, despite having high binding affinity, were incapable of toxin neutralization *in vitro*. One of the isolated sdAb, B39, seemed to have four binding sites for TcdB. Interestingly, B5.2 cross-reacted with the TcdA but was unable to neutralize it.

Noteworthy, an attractive alternative to the combination of antibodies is the use of bispecific or multispecific antibody constructs, which can be easily prepared by rAb technologies (254–256). This approach was previously reported for the design of therapeutic antibodies against several diseases such as cancers, inflammatory and infectious diseases (257, 258). Concerning *C. difficile*, Yang et al. (217) characterized bispecific sdAb antibodies with high efficiency that target both toxins and can effectively treat severe CDI. This work developed several sdAbs that recognized and neutralized either TcdA or TcdB. Additionally, this research group designed a novel construct consisting of multiple-antitoxin sdAbs (AH3–E3–E3–AA6), later called ABA, which neutralized both TcdA and TcdB and reduced disease symptoms in a mouse model of CDI. Evaluation of the efficiency of ABA in the neutralization of the toxin from different clinical *C. difficile* isolates indicated considerable ability of ABA to neutralize toxins in the isolates that produced both TcdA and TcdB, but inefficient to neutralize the toxin from TcdA^-^B^+^ *C. difficile* strains. Importantly, the application of ABA could protect mice against a systemic challenge of a mixture of TcdA and TcdB, indicating its high potency also *in vivo*. The results of this study have proven high therapeutic efficacy of sdAb antibodies against both toxins in multivalent or bispecific formats, through reducing the morbidity and mortality associated with this disease.

In the work reported by Hussack et al. (2018), the stability of sdAb fragments isolated was also increased through fusions to Fc to reach the neutralizing potency of bezlotoxumab in *in vitro* assays. Epitope binding revealed that bivalent sdAb-Fc fusions can target TcdB at regions both similar and distinct from the bezlotoxumab binding sites so that some constructs of sdAb-Fcs could recognize the sites distinct from the binding site of bezlotoxumab and other sdAb-Fcs had binding site similar to bezlotoxumab. Overall, the sdAbs described in this work were effective in toxin B neutralization when provided in bivalent sdAb-Fc formats (259).

The advantage of rAbs is the possibility of their genetic manipulation to increase their efficiency. Sulea et al. (2018) considered an affinity maturation platform to construct a set of mutant sdAbs neutralizing TcdA. These results supported the role of mutation in enhancing the affinity of antibodies. In this regard, the development of double-mutant T56R and T103R neutralized TcdA cytotoxicity with a half maximal inhibitory concentration (IC50) of 12 nM and enhanced sdAb affinity to toxin A (260).

### Probiotic bacteria expressing antitoxin fragments

Recently, the use of probiotic bacteria expressing antibodies has attracted the attention of many researchers. For *C. difficile*, the expression of antibodies on the surface of probiotic bacteria has been investigated in some studies and it was shown that this approach retained neutralizing potency of the antibodies used. In this regard, Andersen et al. (2015) assessed the expression of four anti-toxins sdAb fragments on the surface of *Lactobacillus paracasei* strains. Interestingly, two strains of the probiotic delayed the death of hamsters challenged by *C. difficile* spores, whereas no animal in the control group survived (non-sdAb expressing *Lactobacillus* group vs infection only group). Additionally, 50% of the hamsters receiving the probiotic survived until the end of the experiment. Noteworthy, following administration of purified anti-TcdB VHH alone, no protection was observed *in vivo* that was probably related to the degradation of the antibodies in the GI tract. In fact, the expression of antibodies on the surface of *Lactobacilli* helps preserve antibodies in the GI tract (218). Also, Shkoporov et al. (2015) described the expression of two sdAbs on the surface of *Bifidobacterium longum* and showed TcdA neutralization *in vitro*. Moreover, administration of recombinant *B. longum* to mice revealed *in vivo* expression of both sdAbs in the gut of mice (261). Recently, Chen et al. (2020) assessed the expression of a single tetra-specific antibody on the surface of *Saccharomyces boulardii*. The results showed that engineered probiotic bacteria neutralized both toxins and demonstrated that it can protect mice in both primary and rCDI models. This study proposed that the co-administration of engineered *S. boulardii* with antibiotics may have potential to be regarded as a therapeutic tool for patients with CDI (262). Based on these results, the application of engineered probiotic bacteria producing surface-exposed anti-toxins can be considered a complementary approach for CDI future treatment.

### Recombinant antibodies to other *C. difficile* targets

In addition to TcdA and TcdB, CDT is an important pathogenicity factor of *C. difficile* that can cause death in animals (263). Unger et al. (2015) introduced and characterized specific sdAbs from phage display libraries generated from immunized llamas. These sdAbs could block enzymatic and cytotoxic activities of CDT. Thus, these sdAbs can be considered as a promising new tool for diagnosis and therapy of CDI (253).

Noteworthy, administration of antitoxin antibodies is effective only in developed CDI and does not prevent the initial *C. difficile* colonization step, thus antibodies that are capable of binding to other virulence factors of *C. difficile* appear attractive as complement tools for many researchers. For example, antibodies against cell-surface components involved in the adherence to host gut tissues and colonization, such as SLPs (264, 265), flagella (266), Cwp84 (267), are other promising complementary targets for antibody therapy. Several studies suggest that the application of anti-SLPs antibodies can be a good choice for CDI treatment (268, 269). For instance, anti-SLP sdAbs could inhibit bacterial motility in *in vitro* assays (219). The sdAbs demonstrated broad binding specificity to different *C. difficile* RTs, including RT001, RT027, RT012, RT017, RT023, and RT078. These results showed that targeting SLPs with rAbs should be considered in antibody-mediated therapy for CDI.

Several studies have also proposed the use of anti-flagella antibodies as therapeutics (268, 270). There is one report on the isolation of scFv fragments against FliC and FliD of *C. difficile* that inhibited bacterial motility (220). According to this, it seems that targeting surface components of *C. difficile* by antibodies can be of therapeutic value, thus targeting components such as FliC and FliD, Cwp84, and PSII polysaccharides could be appraised in the development of antibodies in the future.

## Discussion

Today, the use of antibodies for therapeutic purposes has received much attention. In this regard, obtaining antibodies with specificity, sensitivity and high affinity has always been a challenge for researchers. Recently, many efforts have been made to achieve specific antibodies against *C. difficile* proteins for therapeutic purposes, and a considerable amount of research is still ongoing in this area. It has been proven that rAb fragments, especially sdAbs are potentially effective tools for therapy of *C. difficile* (271, 272). In most cases, rAbs have many advantages, including the ability to be genetically modified to improve selectivity, sensitivity and immobilization, thus they have been proposed as alternatives to conventional antibodies.

Recently, rAbs have been widely studied for clinical applications. Antibody therapy has been considered in GI diseases as an efficient method. Hence, achievement of high affinity antibodies that can compete with other treatments such as antibiotics is of great importance in clinical setting. The use of rAbs for therapeutic purposes has become popular in recent years because these antibodies can overcome many of the disadvantages of conventional immunotherapy methods. In a clinical trial, systemic administration of llama-derived nanobodies by intravenous or subcutaneous injections to more than 700 humans produced no adverse side effects (273). Moreover, local administration of rAbs to the GI tract, e.g. by oral or rectal administration, has been proposed. In this case, encapsulation of rAbs can be useful to protect them from damage caused by low gastric pH and pancreatic proteases (274). Furthermore, rectal administration, e.g. as a supplement to fecal microbiome transplantation (FMT), may also be possible (253, 275–277). For this purpose, immobilization of rAbs on beads may help their absorption and eliminate soluble toxins through the rectum.

Another advantage of using rAbs is that they can be used in the form of bispecific or multi-specific antibodies. Bispecific antibodies often maintain the properties of their parental antibodies, but they are more effective. Additionally, production of bispecific or multi-specific antibodies is of low cost and is preferred over combination therapies. In fact, the number of antibodies required to be developed is reduced through production of bispecific or multi-specific antibodies. Interestingly, a limitation of rAbs is their short half-life compared to mAbs, which can be increased by simple strategies such as genetic fusion to an albumin-specific nanobody and genetic fusion to the Fc domain of a conventional IgG antibody (278, 279). These strategies can protect rAbs from intracellular degradation (49, 129, 280, 281), and as a result, these methods are useful in prolonging the life of rAb fragments. However, bispecific or multi-specific rAbs may not need this reinforcement as their size confers sufficient *in vivo* half-life. In case of a bispecific tetramer designed based on an anti-toxin A and B, it was shown that this tetramer protects against death in CDI mice (217). This result confirmed the effectivity of these bispecific antibodies in reducing the severity of CDI and supports the hypothesis that increases in the size of sdAbs by using fusion constructs can improve the neutralization potency of sdAbs. Another major consideration is that the integration of protein engineering and rAbs technologies has led to the development of antibodies resistant to stomach acid and GI proteases. The use of different strategies, e.g. masking protease active sites, enzyme inhibition, pH modulation, and encapsulation, can improve the stability of antibodies (108). Additionally, the use of site-directed mutagenesis and genetic engineering can help select rAbs with high thermostability (282). In this regard, the use of disulfide engineering on anti-TcdB sdAbs enhanced thermostability and resistance to acidic pH without reducing their neutralization capabilities (214).

Expression of rAbs on the cell surface or as secretory proteins of *lactobaccili* is another feasible option (283, 284). Displaying rAbs fragments on probiotic bacterial surface is a two-way solution for disease control that helps in maintaining the gut microbiota and can preserve antibodies in the GI tract for a long time (283, 285–287). There are many advantages for this method, including cost-effective production, ease of administration, long shelf life, use of probiotic bacteria as complementary to treatment, and facility of genetic manipulation of bacteria, which make it an interesting topic for many researchers. This method has shown promising results in several GI infections (218). For instance, the use of anti-CDT sdAbs displayed on *lactobacilli* was capable to protect a hamster model of CDI (218). Interestingly, it was suggested that probiotic bacteria like *B. longum* have a much higher efficiency in secreting sdAbs in a functional form than scFvs (216).

Another key point is the possibility of using the sequence of these antibodies as an option in gene therapy. Gene therapy based on bispecific sdAb fragments against TcdA and TcdB could effectively neutralize toxins in animal models of CDI (217), and it is speculated to receive more attention in future research. Additionally, the application of scRNA-seq technologies for constructing or screening display libraries can help select antibodies with functional properties and high affinity (154, 155). These technologies can efficiently preserve the cognate V_H_–V_L_ pairing and *in vivo* maturation of antibodies, thus will be further considered in the field of antibody therapy in the near future (159, 160). Overall, the effectiveness of rAbs can easily be enhanced by various methods and they can be employed as alternative therapeutics in future (260). We expect therefore to achieve practical information on desirable properties, efficacy and clinical applications of rAbs in the coming years.

## Conclusion

Taken together, both mAb and rAb fragments (i.e., scFv and sdAb) are capable of CDI immunotherapy. However, a better understanding of *C. difficile* biology and the role of its virulence factors would help targeted treatment of severe CDI or rCDI caused by hypervirulent strains. Previous studies have demonstrated that rAbs, especially scFvs and sdAbs, have low molecular weight, high antigen affinity, good stability, and fast tissue penetration, which lead to their extremely wide applications in diagnosis, treatment, and prevention of diseases. As a result, these antibodies can be a reliable option for therapeutic purposes. In our opinion, the future trends and upcoming research on the development of specific antibodies for CDI treatment will focus on application of high-specificity biomolecules such as rAbs, especially the use of probiotic bacteria expressing rAbs, which can also maintain and improve the diversity and integrity of the gut microbiome. Additionally, integration of antibody therapy with FMT may augment the gut microbiota normalization of recipients and increase the efficiency of fecal transplant. Further investigations are needed to meet these claims and provide important information regarding the application of rAbs-based treatments for clinicians and patients with CDI.

## Author contributions

HR was involved in writing of the original draft and constructing tables. MA and AN-R were involved in constructing figures and conceptualization. AY was involved in preparing the draft of the manuscript, reviewing, and editing. HAA and MRZ were involved in reviewing and revising the manuscript. All authors contributed to the article and approved the submitted version.

## Funding

This study was financially supported by a research grant (no. RIGLD 1138, IR.SBMU.RIGLD.REC.1399.051) from the Foodborne and Waterborne Diseases Research Center, Research Institute for Gastroenterology and Liver Diseases, Shahid Beheshti University of Medical Sciences, Tehran, Iran.

## Acknowledgments

The authors would like to thank the members of the Foodborne and Waterborne Diseases Research Center at the Research Institute for Gastroenterology and Liver Diseases, Shahid Beheshti University of Medical Sciences, Tehran, Iran.

## Conflict of interest

The authors declare that the research was conducted in the absence of any commercial or financial relationships that could be construed as a potential conflict of interest.

## Publisher’s note

All claims expressed in this article are solely those of the authors and do not necessarily represent those of their affiliated organizations, or those of the publisher, the editors and the reviewers. Any product that may be evaluated in this article, or claim that may be made by its manufacturer, is not guaranteed or endorsed by the publisher.

## Abbreviations

AAD, Antibiotic-associated diarrhea; Cwps, Cell wall proteins; *C. difficile*, *Clostridioides difficile*; CDI, *C. difficile* infection; cDNA, Complementary DNA; CROPs, Combined repetitive oligopeptide structures; CDRs, Complementarity determining regions; EMA, European Medicines Agency; Fabs, Fragment antigen binding domains; Fc, Fragment crystallizable; FACS, Fluorescence-activated cell sorting; FDA, Food and Drug Administration; FliC, Flagellin; FliD, Flagellin filament cap proteins; FR, Framework regions; FMT, Fecal microbiome transplantation; GI tract, Gastrointestinal tract; GTD, Glucosyltransferase domain; IC50, Half maximal inhibitory concentration; HAMA, Human anti-mouse antibody reaction; HIV-1, Human immunodeficiency virus type 1, HuMAbs, Human monoclonal IgGs; Ig, Immunoglobulin; IGIV, Immune globulin intravenous; mAbs, Monoclonal antibodies; NGS, Next-generation sequencing; PaLoc, Pathogenicity locus; PD-1, Programmed cell death protein 1; pAbs, Polyclonal antibodies; PMC, Pseudomembranous colitis; RA, Rheumatoid arthritis; rAbs, Recombinant antibodies; rCDI, Recurrent CDI; SLP, S-layer proteins; scFv, Single-chain fragment variable; scRNA-seq, Single-cell RNA sequencing technologies, sdAb, Single-domain antibodies; SoC, Standard of care; TLRs, Toll-like receptors; TNFα, Tumor necrosis factor α; VEGF-A, Vascular endothelial growth factor A; V_H_, Variable regions of heavy domain; V_L_, Variable regions of light domain.

## References

[1] Tedesco FJ. Clindamycin-associated colitis. Ann Internal Med (1974) 81:492. doi: 10.7326/0003-4819-81-4-429 4412460

[2] Yassin S Young-Fadok T Zein N Pardi D. Clostridium difficile-associated diarrhea and colitis. Mayo Clinic Proc Mayo Clinic (2001) 76:725–30. doi: 10.4065/76.7.725 11444405

[3] Padua D Pothoulakis C. Novel approaches to treating clostridium difficile-associated colitis. Expert Rev Gastroenterol Hepatology (2016) 10(2):193–204. doi: 10.1586/17474124.2016.1109444 PMC5979265 26643655

[4] Warny M Pepin J Fang A Killgore G Thompson A Brazier J. Toxin production by an emerging strain of clostridium difficile associated with outbreaks of severe disease in north America and Europe. Lancet (2005) 366:1079–84. doi: 10.1016/S0140-6736(05)67420-X 16182895

[5] Chilton CH Pickering DS Freeman J. Microbiologic factors affecting clostridium difficile recurrence. Clin Microbiol Infect (2018) 24(5):476–82. doi: 10.1016/j.cmi.2017.11.017 29208562

[6] Smits WK Lyras D Lacy DB Wilcox MH Kuijper EJ. Clostridium difficile infection. Nat Rev Dis Primers (2016) 2:16020. doi: 10.1038/nrdp.2016.20 27158839 PMC5453186

[7] Isidro J Mendes A Serrano M Henriques A Oleastro M. Overview of clostridium difficile infection: Life cycle, epidemiology, antimicrobial resistance and treatment. Enany S. Clostridium Difficile - A Comprehensive Overview. London:IntechOpen (2017). doi: 10.5772/intechopen.69053

[8] Azimirad M Noori M Raeisi H Yadegar A Shahrokh S Asadzadeh Aghdaei H. How does COVID-19 pandemic impact on incidence of clostridioides difficile infection and exacerbation of its gastrointestinal symptoms? Front Med (2021) 8. doi: 10.3389/fmed.2021.775063 PMC8710593 34966759

[9] Rineh A Kelso MJ Vatansever F Tegos GP Hamblin MR. Clostridium difficile infection: molecular pathogenesis and novel therapeutics. Expert Rev Anti-infective Ther (2014) 12(1):131–50. doi: 10.1586/14787210.2014.866515 PMC4306399 24410618

[10] Crobach MJT Vernon JJ Loo VG Kong LY Péchiné S Wilcox MH. Understanding clostridium difficile colonization. Clin Microbiol Rev (2018) 31(2). doi: 10.1128/CMR.00021-17 PMC5967689 29540433

[11] Voth DE Ballard JD. Clostridium difficile toxins: mechanism of action and role in disease. Clin Microbiol Rev (2005) 18(2):247–63. doi: 10.1128/CMR.18.2.247-263.2005 PMC1082799 15831824

[12] Di Bella S Ascenzi P Siarakas S Petrosillo N Di Masi A. Clostridium difficile toxins a and b: Insights into pathogenic properties and extraintestinal effects. Toxins (2016) 8:134. doi: 10.3390/toxins8050134 PMC4885049 27153087

[13] Stranges P Hutton D Collins C. Cost-effectiveness analysis evaluating fidaxomicin versus oral vancomycin for the treatment of clostridium difficile infection in the united states. Value Health J Int Soc Pharmacoeconomics Outcomes Res (2013) 16:297–304. doi: 10.1016/j.jval.2012.11.004 23538181

[14] Cornely O Miller M Louie T Crook D. Treatment of first recurrence of clostridium difficile infection: Fidaxomicin versus vancomycin. Clin Infect Dis An Off Publ Infect Dis Soc America (2012) 55 Suppl 2:S154–61. doi: 10.1093/cid/cis462 PMC3388030 22752865

[15] Czepiel J Dróżdż M Pituch H Kuijper EJ Perucki W Mielimonka A. Clostridium difficile infection: review. Eur J Clin Microbiol Infect Dis (2019) 38(7):1211–21. doi: 10.1007/s10096-019-03539-6 PMC6570665 30945014

[16] Mullane K. Fidaxomicin in clostridium difficile infection: Latest evidence and clinical guidance. Ther Adv Chronic Disease (2014) 5:69–84. doi: 10.1177/2040622313511285 PMC3926343 24587892

[17] Wilcox M Gerding D Poxton I Kelly C Nathan R Birch T. Bezlotoxumab for prevention of clostridium difficile infection recurrence. New Engl J Med (2016) 376:305–17. doi: 10.1056/NEJMoa1602615 28121498

[18] Lowy I Molrine D Leav B Blair B Baxter R Gerding D. Treatment with monoclonal antibodies against clostridium difficile toxins. New Engl J Med (2010) 362:197–205. doi: 10.1056/NEJMoa0907635 20089970

[19] Davies N Compson J Mackenzie B O'Dowd V Oxbrow A Heads J. A mixture of functionally oligoclonal humanized monoclonal antibodies that neutralize clostridium difficile TcdA and TcdB with high levels of *In vitro* potency shows *In vivo* protection in a hamster infection model. Clin Vaccine Immunol CVI (2013) 20:377–90 doi: 10.1128/CVI.00625-12 PMC3592348 23324518

[20] Wilcox M. Descriptive study of intravenous immunoglobulin for the treatment of recurrent clostridium difficile diarrhoea. J Antimicrobial Chemo (2004) 53:882–4. doi: 10.1093/jac/dkh176 15073160

[21] Saito T Kimura S Tateda K Mori N Hosono N Hayakawa K. Evidence of intravenous immunoglobulin as a critical supportive therapy against clostridium difficile toxin-mediated lethality in mice. J Antimicrobial Chemo (2011) 66:1096–9. doi: 10.1093/jac/dkr027 21393125

[22] Sougioultzis S Kyne L Drudy D Keates S Maroo S Pothoulakis C. Clostridium difficile toxoid vaccine in recurrent c. difficile-associated diarrhea. Gastroenterology (2005) 128:764–70. doi: 10.1053/j.gastro.2004.11.004 15765411

[23] Leuzzi R Adamo R Scarselli M. Vaccines against clostridium difficile. Hum Vaccines Immunotherapeutics (2014) 10:1466–77. doi: 10.4161/hv.28428 PMC5396221 24637887

[24] Förster B Chung P Crobach M Kuijper E. Application of antibody-mediated therapy for treatment and prevention of clostridium difficile infection. Front Microbiol (2018) 9:1382. doi: 10.3389/fmicb.2018.01382 29988597 PMC6027166

[25] Humphreys DP Wilcox MH. Antibodies for treatment of clostridium difficile infection. Clin Vaccine Immunol CVI (2014) 21(7):913–23. doi: 10.1128/CVI.00116-14 PMC4097441 24789799

[26] Baines SD Wilcox MH. Antimicrobial resistance and reduced susceptibility in clostridium difficile: Potential consequences for induction, treatment, and recurrence of c. difficile infection. Antibiotics (2015) 4(3):267–98. doi: 10.3390/antibiotics4030267 PMC4790285 27025625

[27] Citron DM Merriam CV Tyrrell KL Warren YA Fernandez H Goldstein EJ. *In vitro* activities of ramoplanin, teicoplanin, vancomycin, linezolid, bacitracin, and four other antimicrobials against intestinal anaerobic bacteria. Antimicrob Agents Chemother (2003) 47(7):2334–8. doi: 10.1128/AAC.47.7.2334-2338.2003 PMC161871 12821492

[28] Goldstein EJ Babakhani F Citron DM. Antimicrobial activities of fidaxomicin. Clin Infect Dis (2012) 55 Suppl 2(Suppl 2):S143–8. doi: 10.1093/cid/cis339 PMC3388021 22752863

[29] Theriot CM Koenigsknecht MJ Carlson PE Hatton GE Nelson AM Li B. Antibiotic-induced shifts in the mouse gut microbiome and metabolome increase susceptibility to clostridium difficile infection. Nat Commun (2014) 5(1):3114. doi: 10.1038/ncomms4114 24445449 PMC3950275

[30] Johanesen PA Mackin KE Hutton ML Awad MM Larcombe S Amy JM. Disruption of the gut microbiome: Clostridium difficile infection and the threat of antibiotic resistance. Genes (Basel) (2015) 6(4):1347–60. doi: 10.3390/genes6041347 PMC4690045 26703737

[31] Deshpande A Pasupuleti V Thota P Pant C Rolston DD Sferra TJ. Community-associated clostridium difficile infection and antibiotics: a meta-analysis. J Antimicrob Chemother (2013) 68(9):1951–61. doi: 10.1093/jac/dkt129 23620467

[32] Kelly CP. Can we identify patients at high risk of recurrent clostridium difficile infection? Clin Microbiol Infect (2012) 18:21–7. doi: 10.1111/1469-0691.12046 23121551

[33] Song JH Kim YS. Recurrent clostridium difficile infection: Risk factors, treatment, and prevention. Gut Liver (2019) 13(1):16–24. doi: 10.5009/gnl18071 PMC6346998 30400734

[34] Ghosh S. Monoclonal antibodies: A tool in clinical research. Indian J Clin Med (2013) 2013:9–21. doi: 10.4137/IJCM.S11968

[35] Siddiqui M. Monoclonal antibodies as diagnostics; an appraisal. Indian J Pharm Sci (2010) 72:12–7. doi: 10.4103/0250-474X.62229 PMC2883214 20582184

[36] Haji-Hashemi H Norouzi P Safarnejad MR Larijani B Habibi MM Raeisi H. Sensitive electrochemical immunosensor for citrus bacterial canker disease detection using fast Fourier transformation square-wave voltammetry method. J Electroanalytical Chem (2018) 820:111–7. doi: 10.1016/j.jelechem.2018.04.062

[37] Raeisi H Safarnejad MR Alavi SM Farrokhi N Elahinia SA Safarpour H. Development and molecular analyses of xanthomonas pthA specific scFv recombinant monoclonal antibodies. mdrsjrns (2019) 8(4):417–29.

[38] Raeisi H Safarnejad MR Alavi SM Farrokhi N Elahinia SA. Transient expression of an scFvG8 antibody in plants and characterization of its effects on the virulence factor pthA of xanthomonas citri subsp. citri. Transgenic Res (2022) 31(2):269–83. doi: 10.1007/s11248-022-00301-1 35237898

[39] Alibeiki M Golchin M Tabatabaei M. Development of a double-recombinant antibody sandwich ELISA for quantitative detection of epsilon toxoid concentration in inactivated clostridium perfringens vaccines. BMC Veterinary Res (2020) 16:361. doi: 10.1186/s12917-020-02572-4 PMC7525996 32993643

[40] Kim H-Y Lee J-H Kim M Park S Choi M Lee W. Development of a SARS-CoV-2-specific biosensor for antigen detection using scFv-fc fusion proteins. Biosensors Bioelectronics (2020) 175:112868. doi: 10.1016/j.bios.2020.112868 33281048 PMC7703470

[41] Chames P Van Regenmortel M Weiss E Baty D. Therapeutic antibodies: Successes, limitations and hopes for the future. Br J Pharmacol (2009) 157:220–33. doi: 10.1111/j.1476-5381.2009.00190.x PMC2697811 19459844

[42] Wilcox MH Gerding DN Poxton IR Kelly C Nathan R Birch T. Bezlotoxumab for prevention of recurrent clostridium difficile infection. N Engl J Med (2017) 376(4):305–17. doi: 10.1056/NEJMoa1602615 28121498

[43] Lamore R 3rd Parmar S Patel K Hilas O. Belimumab (benlysta): a breakthrough therapy for systemic lupus erythematosus. P T (2012) 37(4):212–26. PMC3351861 22593633

[44] Mazumdar S. Raxibacumab. mAbs (2009) 1(6):531–8. doi: 10.4161/mabs.1.6.10195 PMC2791309 20068396

[45] Tamilarasan AG Cunningham G Irving PM Samaan MA. Recent advances in monoclonal antibody therapy in IBD: practical issues. Frontline Gastroenterol (2019) 10(4):409–16. doi: 10.1136/flgastro-2018-101054 PMC6788124 31656567

[46] Siegel D. Recombinant monoclonal antibody technology. Transfusion clinique biologique J la Société Française Transfusion Sanguine (2002) 9:15–22. doi: 10.1016/S1246-7820(01)00210-5 11889896

[47] Ch'ng ACW Choong YS Lim TS. Phage display-derived antibodies: Application of recombinant antibodies for diagnostics. Saxena SK, Proof and Concepts in Rapid Diagnostic Tests and Technologies. London:IntechOpen (2016). 10.5772/63927

[48] Valldorf B Hinz SC Russo G Pekar L Mohr L Klemm J. Antibody display technologies: selecting the cream of the crop. Biol Chem (2022) 403(5-6):455–77. doi: 10.1515/hsz-2020-0377 33759431

[49] Ahmad A Yeap SK Ali A Ho WY Alitheen N Hamid M. scFv antibody: Principles and clinical application. Clin Dev Immunol (2012) 2012:980250. doi: 10.1155/2012/980250 22474489 PMC3312285

[50] Kallewaard NL Corti D Collins PJ Neu U McAuliffe JM Benjamin E. Structure and function analysis of an antibody recognizing all influenza a subtypes. Cell (2016) 166(3):596–608. doi: 10.1016/j.cell.2016.05.073 PMC4967455 27453466

[51] Kunik V Peters B Ofran Y. Structural consensus among antibodies defines the antigen binding site. PLoS Comput Biol (2012) 8(2):e1002388. doi: 10.1371/journal.pcbi.1002388 22383868 PMC3285572

[52] Melarkode Vattekatte A Shinada NK Narwani TJ Noël F Bertrand O Meyniel JP. Discrete analysis of camelid variable domains: sequences, structures, and in-silico structure prediction. PeerJ (2020) 8:e8408. doi: 10.7717/peerj.8408 32185102 PMC7061911

[53] Chiu M Goulet D Teplyakov A Gilliland G. Antibody structure and function: The basis for engineering therapeutics. Antibodies (2019) 8:55. doi: 10.3390/antib8040055 PMC6963682 31816964

[54] Dondelinger M Filée P Sauvage E Quinting B Muyldermans S Galleni M. Understanding the significance and implications of antibody numbering and antigen-binding Surface/Residue definition. Front Immunol (2018) 9. doi: 10.3389/fimmu.2018.02278 PMC6198058 30386328

[55] Lipman N Jackson L Trudel L Weis-Garcia F. Monoclonal versus polyclonal antibodies: Distinguishing characteristics, applications, and information resources. ILAR J / Natl Res Council Institute Lab Anim Resources (2005) 46:258–68. doi: 10.1093/ilar.46.3.258 15953833

[56] Kaplon H Reichert J. Antibodies to watch in 2021. mAbs (2021) 13:1860476. doi: 10.1080/19420862.2020.1860476 33459118 PMC7833761

[57] Parray H Shukla S Samal S Shrivastava T Ahmed S Sharma C. Hybridoma technology a versatile method for isolation of monoclonal antibodies, its applicability across species, limitations, advancement and future perspectives. Int Immunopharmacol (2020) 85. doi: 10.1016/j.intimp.2020.106639 PMC7255167 32473573

[58] Biller LH Schrag D. Diagnosis and treatment of metastatic colorectal cancer: A review. JAMA (2021) 325(7):669–85. doi: 10.1001/jama.2021.0106 33591350

[59] Lopes dos Santos M Quintilio W Manieri T Tsuruta L Moro A. Advances and challenges in therapeutic monoclonal antibodies drug development. Braz J Pharm Sci (2018) 54. doi: 10.1590/s2175-97902018000001007

[60] Köhler G Milstein C. Continuous cultures of fused cells secreting antibody of predefined specificity. Nature (1975) 256(5517):495–7. doi: 10.1038/256495a0 1172191

[61] Lu R-M Hwang Y-C Liu IJ Lee C-C Tsai H-Z Li H-J. Development of therapeutic antibodies for the treatment of diseases. J Biomed Sci (2020) 27. doi: 10.1186/s12929-019-0592-z PMC6939334 31894001

[62] Mendez M Green L Corvalan J Jia X-C Maynard-Currie C Yang X-D. Functional transplant of megabase human immunoglobulin loci recapitulates human antibody response in mice. Nat Genet (1997) 15:146–56. doi: 10.1038/ng0297-146 9020839

[63] Berger M Shankar V Vafai A. Therapeutic applications of monoclonal antibodies. Am J Med Sci (2002) 324:14–30. doi: 10.1097/00000441-200207000-00004 PMC7093874 12120821

[64] Laffleur B Pascal V Sirac C Cogne M. Production of human or humanized antibodies in mice. Methods Mol Biol (2012) 901:149–59. doi: 10.1007/978-1-61779-931-0_9 22723099

[65] Tonegawa S Brack C Hozumi N Schuller R. Cloning of an immunoglobulin variable region gene from mouse embryo. Proc Natl Acad Sci United States America (1977) 74(8):3518–22. doi: 10.1073/pnas.74.8.3518 PMC431622 410021

[66] Boulianne GL Hozumi N Shulman MJ. Production of functional chimaeric mouse/human antibody. Nature (1984) 312(5995):643–6. doi: 10.1038/312643a0 6095115

[67] Almagro J Daniels-Wells T Perez-Tapia S Penichet M. Progress and challenges in the design and clinical development of antibodies for cancer therapy. Front Immunol (2018) 8:1751. doi: 10.3389/fimmu.2017.01751 29379493 PMC5770808

[68] Krauss J. Recombinant antibodies for the diagnosis and treatment of cancer. Mol Biotechnol (2003) 25(1):1–17. doi: 10.1385/MB:25:1:1 13679630

[69] Qu Z Griffiths G Wegener W Chang C-H Govindan S Horak I. Development of humanized antibodies as cancer therapeutics. Methods (2005) 36:84–95. doi: 10.1016/j.ymeth.2005.01.008 15848077

[70] Waldmann H. Human monoclonal antibodies: The benefits of humanization. Methods Mol Biol (2019) 1904:1–10. doi: 10.1007/978-1-4939-8958-4_1 30539464

[71] Roberts S Cheetham J Rees A. Generation of an antibody with enhanced affinity and specificity for its antigen by protein engineering. Nature (1987) 328:731–4. doi: 10.1038/328731a0 3614380

[72] Vaswani S Hamilton R. Humanized antibodies as potential therapeutic drugs. Ann Allergy Asthma Immunol (1998) 81:105–15. doi: 10.1016/S1081-1206(10)62794-9 9723555

[73] Tong X-M Feng L Suthe SR Weng T-H Hu C-Y Liu Y-Z. Therapeutic efficacy of a novel humanized antibody-drug conjugate recognizing plexin-semaphorin-integrin domain in the RON receptor for targeted cancer therapy. J ImmunoTherapy Cancer (2019) 7(1):250. doi: 10.1186/s40425-019-0732-8 PMC6743155 31519211

[74] Gui X Deng M Song H Chen Y Xie J Li Z. Disrupting LILRB4/APOE interaction by an efficacious humanized antibody reverses T-cell suppression and blocks AML development. Cancer Immunol Res (2019) 7:1244–57. doi: 10.1158/2326-6066.CIR-19-0036 PMC6677629 31213474

[75] Dattola A Silvestri M Tamburi F Amoruso G Bennardo L Nisticò S. Emerging role of anti-IL23 in the treatment of psoriasis: When humanized is very promising. Dermatologic Ther (2020) 33:e14504. doi: 10.1111/dth.14504 33141505

[76] Alt F Blackwell TK Yancopoulos G. Immunoglobulin genes in transgenic mice. Trends Genet - Trends Genet (1985) 1:231–6. doi: 10.1016/0168-9525(85)90089-7

[77] Green LL Hardy MC Maynard-Currie CE Tsuda H Louie DM Mendez MJ. Antigen–specific human monoclonal antibodies from mice engineered with human ig heavy and light chain YACs. Nat Genet (1994) 7(1):13–21. doi: 10.1038/ng0594-13 8075633

[78] Brüggemann M Caskey HM Teale C Waldmann H Williams GT Surani MA. A repertoire of monoclonal antibodies with human heavy chains from transgenic mice. Proc Natl Acad Sci United States America (1989) 86:6709–13. doi: 10.1073/pnas.86.17.6709 PMC297915 2505258

[79] Lonberg N. Human monoclonal antibodies from transgenic mice. Handb Exp Pharmacol (2008) 181:69–97. doi: 10.1007/978-3-540-73259-4_4 PMC7120671 18071942

[80] Sergeeva A Kolonin M Molldrem J Pasqualini R Arap W. Display technologies: Application for the discovery of drug and gene delivery agents. Advanced Drug delivery Rev (2007) 58:1622–54. doi: 10.1016/j.addr.2006.09.018 PMC1847402 17123658

[81] Chan SK Rahumatullah A Lai JY Lim TS. Naïve human antibody libraries for infectious diseases. Adv Exp Med Biol (2017) 1053:35–59. doi: 10.1007/978-3-319-72077-7_3 PMC7120739 29549634

[82] Hammers C Stanley J. Antibody phage display: Technique and applications. J Invest Dermatol (2014) 134:e17. doi: 10.1038/jid.2013.521 PMC3951127 24424458

[83] Omar N Lim TS. Construction of naive and immune human fab phage-display library. Methods Mol Biol (2018) 1701:25–44. doi: 10.1007/978-1-4939-7447-4_2 29116498

[84] Willats W. Phage display: Practicalities and prospects. Plant Mol Biol (2003) 50:837–54. doi: 10.1023/A:1021215516430 12516857

[85] Yan J Li G Hu Y Ou W Wan Y. Construction of a synthetic phage-displayed nanobody library with CDR3 regions randomized by trinucleotide cassettes for diagnostic applications. J Trans Med (2014) 12(1):343. doi: 10.1186/s12967-014-0343-6 PMC4269866 25496223

[86] Alfaleh MA Alsaab HO Mahmoud AB Alkayyal AA Jones ML Mahler SM. Phage display derived monoclonal antibodies: From bench to bedside. Front Immunol (2020) 11. doi: 10.3389/fimmu.2020.01986 PMC7485114 32983137

[87] Rami A Behdani M Yardehnavi N Habibi-Anbouhi M Kazemi-Lomedasht F. An overview on application of phage display technique in immunological studies. Asian Pacific J Trop Biomedicine (2017) 7(7):599–602. doi: 10.1016/j.apjtb.2017.06.001

[88] Lim CC Choong YS Lim TS. Cognizance of molecular methods for the generation of mutagenic phage display antibody libraries for affinity maturation. Int J Mol Sci (2019) 20(8). doi: 10.3390/ijms20081861 PMC6515083 30991723

[89] Sheehan J Marasco WA. Phage and yeast display. Microbiol Spectr (2015) 3(1). doi: 10.1128/microbiolspec.AID-0028-2014 26104550

[90] Wang Y Li X Chen X Nielsen J Petranovic D Siewers V. Expression of antibody fragments in saccharomyces cerevisiae strains evolved for enhanced protein secretion. Microbial Cell Factories (2021) 20(1):134. doi: 10.1186/s12934-021-01624-0 34261490 PMC8278646

[91] Liu Y Huang H. Expression of single-domain antibody in different systems. Appl Microbiol Biotechnol (2018) 102(2):539–51. doi: 10.1007/s00253-017-8644-3 29177623

[92] Holliger P Hudson P. Engineered antibody fragments and the rise of single domains. Nat Biotechnol (2005) 23:1126–36. doi: 10.1038/nbt1142 16151406

[93] Basu K Green EM Cheng Y Craik CS. Why recombinant antibodies — benefits and applications. Curr Opin Biotechnol (2019) 60:153–8. doi: 10.1016/j.copbio.2019.01.012 PMC6728236 30849700

[94] Gray A Bradbury ARM Knappik A Plückthun A Borrebaeck CAK Dübel S. Animal-free alternatives and the antibody iceberg. Nat Biotechnol (2020) 38(11):1234–9. doi: 10.1038/s41587-020-0687-9 33046876

[95] Furuta M Uchikawa M Ueda Y Yabe T Taima T Tsumoto K. Construction of mono- and bivalent human single-chain fv fragments against the d antigen in the Rh blood group: Multimerization effect on cell agglutination and application to blood typing. Protein Eng (1998) 11:233–41. doi: 10.1093/protein/11.3.233 9613848

[96] Petrov K Dion M Hoffmann L Dintinger T Defontaine A Tellier C. Bivalent fv antibody fragments obtained by substituting the constant domains of a fab fragment with heterotetrameric molybdopterin synthase. J Mol Biol (2004) 341:1039–48. doi: 10.1016/j.jmb.2004.06.075 15328616

[97] Hudson P Kortt A. High avidity scFv multimers; diabodies and triabodies. J Immunol Methods (2000) 231:177–89. doi: 10.1016/S0022-1759(99)00157-X 10648937

[98] Cuesta A Sainz-Pastor N Bonet J Oliva B Alvarez-Vallina L. Multivalent antibodies: When design surpasses evolution. Trends Biotechnol (2010) 28:355–62. doi: 10.1016/j.tibtech.2010.03.007 20447706

[99] Høydahl LS Nilssen NR Gunnarsen KS MFd Pré Iversen R Roos N. Multivalent pIX phage display selects for distinct and improved antibody properties. Sci Rep (2016) 6(1):39066. doi: 10.1038/srep39066 27966617 PMC5155289

[100] Bazan J Calkosinski I Gamian A. Phage display–a powerful technique for immunotherapy. Hum Vaccines Immunotherapeutics (2012) 8:1829–35. doi: 10.4161/hv.21704 PMC3656072 22906938

[101] Frenzel A Hust M Schirrmann T. Expression of recombinant antibodies. Front Immunol (2013) 4:217. doi: 10.3389/fimmu.2013.00217 23908655 PMC3725456

[102] Shali A Hasannia S Gashtasbi F Shahangian S Jalili S. Generation and screening of efficient neutralizing single domain antibodies (VHHs) against the critical functional domain of anthrax protective antigen (PA). Int J Biol Macromolecules (2018) 114:1267–1278. doi: 10.1016/j.ijbiomac.2018.03.034 29524493

[103] Liu J Ting J Al-Azzam S Ding Y Afshar S. Therapeutic advances in diabetes, autoimmune, and neurological diseases. Int J Mol Sci (2021) 22:2805. doi: 10.3390/ijms22062805 33802091 PMC8001105

[104] Roth K Wenzel E Ruschig M Steinke S Langreder N Heine P. Developing recombinant antibodies by phage display against infectious diseases and toxins for diagnostics and therapy. Front Cell Infect Microbiol (2021) 11:697876. doi: 10.3389/fcimb.2021.697876 34307196 PMC8294040

[105] Kobayashi N Ohtoyo M Wada E Kato Y Mano N Goto J. Generation of a single-chain fv fragment for the monitoring of deoxycholic acid residues anchored on endogenous proteins. Steroids (2005) 70:285–94. doi: 10.1016/j.steroids.2004.11.012 15784283

[106] Tullila A Nevanen T. Utilization of multi-immunization and multiple selection strategies for isolation of hapten-specific antibodies from recombinant antibody phage display libraries. Int J Mol Sci (2017) 18:1169. doi: 10.3390/ijms18061169 PMC5485993 28561803

[107] Guo J-Q You S-Y Li L Zhang Y-Z Huang J-N Zhang C-Y. Erratum to ‘Construction and high-level expression of a single-chain fv antibody fragment specific for acidic isoferritin in escherichia coli’. J Biotechnol (2003) 102:177–89. doi: 10.1016/S0168-1656(03)00020-8 12697395

[108] Jones R Martino A. Targeted localized use of therapeutic antibodies: A review of non-systemic, topical and oral applications. Crit Rev Biotechnol (2016) 36:506–20. 10.3109/07388551.2014.992388 25600465

[109] Sakai K Shimizu Y Chiba T Matsumoto-Takasaki A Kusada Y Zhang W. Isolation and characterization of phage-displayed single chain antibodies recognizing nonreducing terminal mannose residues. 1. a new strategy for generation of anti-carbohydrate antibodies †. Biochemistry (2007) 46:253–62. doi: 10.1021/bi061875e 17198396

[110] Yuasa N Zhang W Goto T Sakaue H Matsumoto-Takasaki A Kimura M. Production of anti-carbohydrate antibodies by phage-display technologies: Potential impairment of cell growth as a result of endogenous expression. J Biol Chem (2010) 285:30587–97. doi: 10.1074/jbc.M110.107284 PMC2945553 20667829

[111] Galeffi P Lombardi A Pietraforte I Novelli F Donato M Sperandei M. Functional expression of a single-chain antibody to ErbB-2 in plants and cell-free systems. J Trans Med (2006) 4:39. doi: 10.1186/1479-5876-4-39 PMC1592514 17010186

[112] Aghebati-Maleki L Younesi V Jadidi-Niaragh F Baradaran B Majidi J Yousefi M. Isolation and characterization of anti ROR1 single chain fragment variable antibodies using phage display technique. Hum Antibodies (2017) 25:1–7. doi: 10.3233/HAB-170310 28128766

[113] Saw PE Song E-W. Phage display screening of therapeutic peptide for cancer targeting and therapy. Protein Cell (2019) 10:787–807. doi: 10.1007/s13238-019-0639-7 PMC6834755 31140150

[114] Lopes R Queiroz M Gomes S Vallinoto AC Goulart L Ishak R. Phage display: an important tool in the discovery of peptides with anti-HIV activity. Biotechnol Advances (2018) 36:1847–1854. doi: 10.1016/j.biotechadv.2018.07.003 30012540

[115] Nixon A Sexton D Ladner R. Drugs derived from phage display. mAbs (2013) 6:73–85. doi: 10.4161/mabs.27240 PMC3929457 24262785

[116] Wesolowski J Alzogaray V Reyelt J Unger M Juarez-Moreno K Urrutia M. Single domain antibodies: Promising experimental and therapeutic tools in infection and immunity. Med Microbiol Immunol (2009) 198:157–74. doi: 10.1007/s00430-009-0116-7 PMC2714450 19529959

[117] Hassanzadeh-Ghassabeh G Devoogdt N De Pauw P Vincke C Muyldermans S. Nanobodies and their potential applications. Nanomedicine (Lond) (2013) 8(6):1013–26. doi: 10.2217/nnm.13.86 23730699

[118] Harmsen M de Haard H. Properties, production, and applications of camelid single-domain antibody fragments. Appl Microbiol Biotechnol (2007) 77:13–22. doi: 10.1007/s00253-007-1142-2 PMC2039825 17704915

[119] Jovcevska I Zupanec N Urlep Ž Vranic A Matos B Limbäck C. Differentially expressed proteins in glioblastoma multiforme identified with a nanobody-based anti-proteome approach and confirmed by OncoFinder as possible tumor-class predictive biomarker candidates. Oncotarget (2017) 8:44141–44158. doi: 10.18632/oncotarget.17390 PMC5546469 28498803

[120] Zhang C Zhang W Tang X Zhang Q Zhang W Li P. Change of amino acid residues in idiotypic nanobodies enhanced the sensitivity of competitive enzyme immunoassay for mycotoxin ochratoxin a in cereals. Toxins (2020) 12(4):273. doi: 10.3390/toxins12040273 PMC7232238 32340239

[121] Muyldermans S. Nanobodies: Natural single-domain antibodies. Annu Rev Biochem (2013) 82:775–797. doi: 10.1146/annurev-biochem-063011-092449 23495938

[122] Siontorou C. Nanobodies as novel agents for disease diagnosis and therapy. Int J Nanomedicine (2013) 8:4215–27. doi: 10.2147/IJN.S39428 PMC3818023 24204148

[123] Salvador JP Vilaplana L Marco MP. Nanobody: outstanding features for diagnostic and therapeutic applications. Analytical Bioanalytical Chem (2019) 411(9):1703–13. doi: 10.1007/s00216-019-01633-4 30734854

[124] Liu J Anderson G Delehanty J Baumann R Hayhurst A Goldman E. Selection of cholera toxin specific IgNAR single-domain antibodies from a naïve shark library. Mol Immunol (2007) 44:1775–83. doi: 10.1016/j.molimm.2006.07.299 17007931

[125] Bradbury AR Sidhu S Dübel S McCafferty J. Beyond natural antibodies: the power of *in vitro* display technologies. Nat Biotechnol (2011) 29(3):245–54. doi: 10.1038/nbt.1791 PMC3057417 21390033

[126] Dyson MR Masters E Pazeraitis D Perera RL Syrjanen JL Surade S. Beyond affinity: selection of antibody variants with optimal biophysical properties and reduced immunogenicity from mammalian display libraries. MAbs (2020) 12(1):1829335. doi: 10.1080/19420862.2020.1829335 33103593 PMC7592150

[127] Bidlingmaier S Su Y Liu B. Combining phage and yeast cell surface antibody display to identify novel cell type-selective internalizing human monoclonal antibodies. Methods Mol Biol (2015) 1319:51–63. doi: 10.1007/978-1-4939-2748-7_3 PMC4842226 26060069

[128] Dreier B Plückthun A. Rapid selection of high-affinity binders using ribosome display. Methods Mol Biol (2012) 805:261–86. doi: 10.1007/978-1-61779-379-0_15 22094811

[129] Frenzel A Kügler J Helmsing S Meier D Schirrmann T Hust M. Designing human antibodies by phage display. Transfusion Med Hemotherapy (2017) 44:312–318. doi: 10.1159/000479633 PMC5649246 29070976

[130] Bakir M Babich J Aftab N Dean C Lambrecht R Ott R. C-erbB2 protein overexpression in breast cancer as a target for PET using iodine-124-labeled monoclonal antibodies. J Nucl Med Off publication Soc Nucl Med (1993) 33:2154–60. 1460508

[131] Yau K Lee H Hall J. Emerging trends in the synthesis and improvement of hapten-specific recombinant antibodies. Biotechnol Advances (2003) 21:599–637. doi: 10.1016/S0734-9750(03)00104-6 14516873

[132] Murdaca G Colombo B Puppo F. Adalimumab for the treatment of immune-mediated diseases: An update on old and recent indications. Drugs Today (2011) 47:277–88. doi: 10.1358/dot.2011.47.4.1576692 21573251

[133] Den Broeder A Putte L Rau R Schattenkirchner M Riel P Sander O. A single dose, placebo controlled study of the fully human anti-tumor necrosis factor-alpha antibody adalimumab (D2E7) in patients with rheumatoid arthritis. J Rheumatol (2002) 29:2288–98. 12415583

[134] Evoy K Abel S. Ranibizumab: The first vascular endothelial growth factor inhibitor approved for the treatment of diabetic macular edema (June). Ann Pharmacotherapy (2013) 47:811–818. doi: 10.1345/aph.1S013 23656749

[135] Ding C. Belimumab, an anti-BLyS human monoclonal antibody for potential treatment of inflammatory autoimmune diseases. Expert Opin Biol Ther (2008) 8:1805–14. doi: 10.1517/14712598.8.11.1805 18847314

[136] Krupitskaya Y Wakelee H. Ramucirumab, a fully human mAb to the transmembrane signaling tyrosine kinase VEGFR-2 for the potential treatment of cancer. Curr Opin Investigational Drugs (2009) 10:597–605. 19513949

[137] Kuenen B Witteveen PO Ruijter R Giaccone G Dontabhaktuni A Fox F. A phase I pharmacologic study of necitumumab (IMC-11F8), a fully human IgG&lt;sub<1&lt;/sub< monoclonal antibody directed against EGFR in patients with advanced solid malignancies. Clin Cancer Res (2010) 16(6):1915. doi: 10.1158/1078-0432.CCR-09-2425 20197484

[138] McDermott D Sosman J Sznol M Massard C Gordon M Hamid O. Atezolizumab, an anti-programmed death-ligand 1 antibody, in metastatic renal cell carcinoma: Long-term safety, clinical activity, and immune correlates from a phase ia study. J Clin Oncol (2016) 34:833–842. doi: 10.1200/JCO.2015.63.7421 26755520

[139] Markham A. Ixekizumab: First global approval. Drugs (2016) 76(8):901–5. doi: 10.1007/s40265-016-0579-y 27098317

[140] Sofen H Smith S Matheson R Leonardi C Calderon C Brodmerkel C. Guselkumab (an IL-23–specific mAb) demonstrates clinical and molecular response in patients with moderate-to-severe psoriasis. J Allergy Clin Immunol (2014) 133:1032–40. doi: 10.1016/j.jaci.2014.01.025 24679469

[141] Boyerinas B Jochems C Fantini M Heery CR Gulley JL Tsang KY. Antibody-dependent cellular cytotoxicity activity of a novel anti–PD-L1 antibody avelumab (MSB0010718C) on human tumor cells. Cancer Immunol Res (2015) 3(10):1148. doi: 10.1158/2326-6066.CIR-15-0059 26014098 PMC4739754

[142] Kenniston JA Faucette RR Martik D Comeau SR Lindberg AP Kopacz KJ. Inhibition of plasma kallikrein by a highly specific active site blocking antibody. J Biol Chem (2014) 289(34):23596–608. doi: 10.1074/jbc.M114.569061 PMC4156074 24970892

[143] Al-Salama Z. Emapalumab: First global approval. Drugs (2019) 79:1–5. doi: 10.1007/s40265-018-1046-8 30623346

[144] Kreitman R Dearden C Zinzani P Delgado J Karlin L Robak T. Moxetumomab pasudotox in relapsed/refractory hairy cell leukemia. Leukemia (2018) 32:1768–1777. doi: 10.1038/s41375-018-0210-1 PMC6087717 30030507

[145] Duggan S. Caplacizumab: First global approval. Drugs (2018) 78(15):1639–42. doi: 10.1007/s40265-018-0989-0 PMC6280848 30298461

[146] Duggan S. Tralokinumab: First approval. Drugs (2021) 81(14):1657–63. doi: 10.1007/s40265-021-01583-1 PMC8519819 34406631

[147] El Debs B Utharala R Balyasnikova IV Griffiths AD Merten CA. Functional single-cell hybridoma screening using droplet-based microfluidics. Proc Natl Acad Sci U S A (2012) 109(29):11570–5. doi: 10.1073/pnas.1204514109 PMC3406880 22753519

[148] Jaroszewicz W Morcinek-Orłowska J Pierzynowska K Gaffke L Węgrzyn G. Phage display and other peptide display technologies. FEMS Microbiol Rev (2021) 46(2). doi: 10.1093/femsre/fuab052 34673942

[149] Li R Kang G Hu M Huang H. Ribosome display: A potent display technology used for selecting and evolving specific binders with desired properties. Mol Biotechnol (2019) 61(1):60–71. doi: 10.1007/s12033-018-0133-0 30406440

[150] Rajan S Kierny MR Mercer A Wu J Tovchigrechko A Wu H. Recombinant human b cell repertoires enable screening for rare, specific, and natively paired antibodies. Commun Biol (2018) 1(1):5. doi: 10.1038/s42003-017-0006-2 30271892 PMC6123710

[151] Nannini F Senicar L Parekh F Kong KJ Kinna A Bughda R. Combining phage display with SMRTbell next-generation sequencing for the rapid discovery of functional scFv fragments. MAbs (2021) 13(1):1864084. doi: 10.1080/19420862.2020.1864084 33382949 PMC7781620

[152] Wang B DeKosky BJ Timm MR Lee J Normandin E Misasi J. Functional interrogation and mining of natively paired human VH:VL antibody repertoires. Nat Biotechnol (2018) 36(2):152–5. doi: 10.1038/nbt.4052 PMC5801115 29309060

[153] Jammes FC Maerkl SJ. How single-cell immunology is benefiting from microfluidic technologies. Microsystems Nanoengineering (2020) 6(1):45. doi: 10.1038/s41378-020-0140-8 34567657 PMC8433390

[154] Seah YFS Hu H Merten CA. Microfluidic single-cell technology in immunology and antibody screening. Mol Aspects Med (2018) 59:47–61. doi: 10.1016/j.mam.2017.09.004 28927942

[155] Wang Y Jin R Shen B Li N Zhou H Wang W. High-throughput functional screening for next-generation cancer immunotherapy using droplet-based microfluidics. Sci Adv (2021) 7(24). doi: 10.1126/sciadv.abe3839 PMC8195480 34117053

[156] Georgiou G Ippolito GC Beausang J Busse CE Wardemann H Quake SR. The promise and challenge of high-throughput sequencing of the antibody repertoire. Nat Biotechnol (2014) 32(2):158–68. doi: 10.1038/nbt.2782 PMC4113560 24441474

[157] Guo Y Huang L Zhang G Yao Y Zhou H Shen S. A SARS-CoV-2 neutralizing antibody with extensive spike binding coverage and modified for optimal therapeutic outcomes. Nat Commun (2021) 12(1):2623. doi: 10.1038/s41467-021-22926-2 33976198 PMC8113581

[158] Rouet R Jackson KJL Langley DB Christ D. Next-generation sequencing of antibody display repertoires. Front Immunol (2018) 9. doi: 10.3389/fimmu.2018.00118 PMC5810246 29472918

[159] Gérard A Woolfe A Mottet G Reichen M Castrillon C Menrath V. High-throughput single-cell activity-based screening and sequencing of antibodies using droplet microfluidics. Nat Biotechnol (2020) 38(6):715–21. doi: 10.1038/s41587-020-0466-7 32231335

[160] Goldstein LD Chen Y-JJ Wu J Chaudhuri S Hsiao Y-C Schneider K. Massively parallel single-cell b-cell receptor sequencing enables rapid discovery of diverse antigen-reactive antibodies. Commun Biol (2019) 2(1):304. doi: 10.1038/s42003-019-0551-y 31428692 PMC6689056

[161] Mazutis L Gilbert J Ung WL Weitz DA Griffiths AD Heyman JA. Single-cell analysis and sorting using droplet-based microfluidics. Nat Protoc (2013) 8(5):870–91. doi: 10.1038/nprot.2013.046 PMC4128248 23558786

[162] Adler AS Mizrahi RA Spindler MJ Adams MS Asensio MA Edgar RC. Rare, high-affinity mouse anti-PD-1 antibodies that function in checkpoint blockade, discovered using microfluidics and molecular genomics. MAbs (2017) 9(8):1270–81. doi: 10.1080/19420862.2017.1371386 PMC5680806 28846506

[163] Asensio MA Lim YW Wayham N Stadtmiller K Edgar RC Leong J. Antibody repertoire analysis of mouse immunization protocols using microfluidics and molecular genomics. MAbs (2019) 11(5):870–83. doi: 10.1080/19420862.2019.1583995 PMC6601537 30898066

[164] Bounab Y Eyer K Dixneuf S Rybczynska M Chauvel C Mistretta M. Dynamic single-cell phenotyping of immune cells using the microfluidic platform DropMap. Nat Protoc (2020) 15(9):2920–55. doi: 10.1038/s41596-020-0354-0 32788719

[165] Rogers TF Zhao F Huang D Beutler N Burns A He WT. Isolation of potent SARS-CoV-2 neutralizing antibodies and protection from disease in a small animal model. Science (2020) 369(6506):956–63. doi: 10.1126/science.abc7520 PMC7299280 32540903

[166] Renn A Fu Y Hu X Hall MD Simeonov A. Fruitful neutralizing antibody pipeline brings hope to defeat SARS-Cov-2. Trends Pharmacol Sci (2020) 41(11):815–29. doi: 10.1016/j.tips.2020.07.004 PMC7572790 32829936

[167] Awad M Johanesen P Carter G Rose E Lyras D. Clostridium difficile virulence factors: Insights into an anaerobic spore-forming pathogen. Gut Microbes (2014) 5:579–593. doi: 10.4161/19490976.2014.969632 PMC4615314 25483328

[168] Azimirad M Krutova M Yadegar A Shahrokh S Olfatifar M Aghdaei HA. Clostridioides difficile ribotypes 001 and 126 were predominant in Tehran healthcare settings from 2004 to 2018: a 14-year-long cross-sectional study. Emerg Microbes Infect (2020) 9(1):1432–43. doi: 10.1080/22221751.2020.1780949 PMC7473134 32520657

[169] Péchiné S Bruxelle J-F Janoir C Collignon A. Targeting clostridium difficile surface components to develop immunotherapeutic strategies against clostridium difficile infection. Front Microbiol (2018) 9. doi: 10.3389/fmicb.2018.01009 PMC5974105 29875742

[170] Azimirad M Naderi Noukabadi F Lahmi F Yadegar A. Prevalence of binary-toxin genes (cdtA and cdtB) among clinical strains of clostridium difficile isolated from diarrheal patients in Iran. Gastroenterol Hepatol Bed Bench (2018) 11(Suppl 1):59–65. PMC6347996 30809324

[171] Poxton IR McCoubrey J Blair G. The pathogenicity of clostridium difficile. Clin Microbiol Infect (2001) 7(8):421–7. doi: 10.1046/j.1198-743x.2001.00287.x 11591205

[172] Natarajan M Walk S Young V. Aronoff d. a clinical and epidemiological review of non-toxigenic clostridium difficile. Anaerobe (2013) 22:1–5. doi: 10.1016/j.anaerobe.2013.05.005 PMC3729612 23727391

[173] Orrell KE Melnyk RA. Large Clostridial toxins: Mechanisms and roles in disease. Microbiol Mol Biol Rev (2021) 85(3):e0006421. doi: 10.1128/MMBR.00064-21 34076506 PMC8483668

[174] Carter GP Rood JI Lyras D. The role of toxin a and toxin b in clostridium difficile-associated disease: Past and present perspectives. Gut Microbes (2010) 1(1):58–64. doi: 10.4161/gmic.1.1.10768 PMC2906822 20664812

[175] Chandrasekaran R Lacy DB. The role of toxins in clostridium difficile infection. FEMS Microbiol Rev (2017) 41(6):723–50. doi: 10.1093/femsre/fux048 PMC5812492 29048477

[176] Monot M Eckert C Lemire A Hamiot A Dubois T Tessier C. Clostridium difficile: New insights into the evolution of the pathogenicity locus. Sci Rep (2015) 5:15023. doi: 10.1038/srep15023 26446480 PMC4597214

[177] Liu Y-W Chen Y-H Chen J-W Tsai P-J Huang I-H. Immunization with recombinant TcdB-encapsulated nanocomplex induces protection against clostridium difficile challenge in a mouse model. Front Microbiol (2017) 8. doi: 10.3389/fmicb.2017.01411 PMC5525027 28790999

[178] Kurtz C Cannon E Brezzani A Pitruzzello M Dinardo C Rinard E. GT160-246, a toxin binding polymer for treatment of clostridium difficile colitis. Antimicrob Agents Chemother (2001) 45:2340–7. doi: 10.1128/AAC.45.8.2340-2347.2001 PMC90651 11451694

[179] Kociolek L Gerding D. Breakthroughs in the treatment and prevention of clostridium difficile infection. Nat Rev Gastroenterol Hepatol (2016) 13:150–160. doi: 10.1038/nrgastro.2015.220 26860266

[180] Yang Z Shi L Yu H Zhang Y Chen K Fleur A. Intravenous adenovirus expressing a multi-specific, single-domain antibody neutralizing TcdA and TcdB protects mice from clostridium difficile infection. Pathog Dis (2016) 74:ftw078. doi: 10.1093/femspd/ftw078 27502696 PMC5985491

[181] Lyerly DM Bostwick EF Binion SB Wilkins TD. Passive immunization of hamsters against disease caused by clostridium difficile by use of bovine immunoglobulin G concentrate. Infect Immun (1991) 59(6):2215–8. doi: 10.1128/iai.59.6.2215-2218.1991 PMC257992 2037383

[182] Kelly C Pothoulakis C Vavva F Castagliuolo I Bostwick E O’Keane C. Anti-clostridium difficile bovine immunoglobulin concentrate inhibits cytotoxicity and enterotoxicity of c. difficile toxins. Antimicrobial Agents Chemo (1996) 40:373–9. doi: 10.1128/AAC.40.2.373 PMC163119 8834883

[183] Kink J Williams J. Antibodies to recombinant clostridium difficile toxins a and b are an effective treatment and prevent relapse of c. difficile-associated disease in a hamster model of infection. Infect Immunity (1998) 66:2018–25. doi: 10.1128/IAI.66.5.2018-2025.1998 PMC108158 9573084

[184] Giannasca P Zhang Z-X Lei W-D Boden J Giel M Monath T. Serum antitoxin antibodies mediate systemic and mucosal protection from clostridium difficile disease in hamsters. Infect Immunity (1999) 67:527–38. doi: 10.1128/IAI.67.2.527-538.1999 PMC96351 9916055

[185] Dissel J Groot N Hensgens C Numan S Kuijper E Veldkamp P. Bovine antibody-enriched whey to aid in the prevention of a relapse of clostridium difficile-associated diarrhoea: preclinical and preliminary clinical data. J Med Microbiol (2005) 54:197–205. doi: 10.1099/jmm.0.45773-0 15673517

[186] O'Brien J McCabe M Athie V McDonald G Eidhin D Kelleher D. Passive immunisation of hamsters against clostridium difficile infection using antibodies to surface layer proteins. FEMS Microbiol Letters (2005) 246:199–205. doi: 10.1016/j.femsle.2005.04.005 15899406

[187] Abougergi M Broor A Cui W Jaar B. Intravenous immunoglobulin for the treatment of severe clostridium difficile colitis: An observational study and review of the literature. J Hosp Med An Off Publ Soc Hosp Med (2010) 5:E1–9. doi: 10.1002/jhm.542 20063275

[188] Mulvey G Dingle T Fang L Strecker J Armstrong G. Therapeutic potential of egg yolk antibodies for treating clostridium difficile infection. J Med Microbiol (2011) 60:1181–7. doi: 10.1099/jmm.0.029835-0 21474614

[189] Roberts A McGlashan J Al-Abdulla I Ling R Denton H Green S. Development and evaluation of an ovine antibody-based platform for treatment of clostridium difficile infection. Infect Immunity (2011) 80:875–82. doi: 10.1128/IAI.05684-11 PMC3264293 22144483

[190] Hutton ML Cunningham BA Mackin KE Lyon SA James ML Rood JI. Bovine antibodies targeting primary and recurrent clostridium difficile disease are a potent antibiotic alternative. Sci Rep (2017) 7(1):3665. doi: 10.1038/s41598-017-03982-5 28623367 PMC5473923

[191] Heidebrecht H-J Weiss W Pulse M Lange A Gisch K Kliem H. Treatment and prevention of recurrent clostridium difficile infection with functionalized bovine antibody-enriched whey in a hamster primary infection model. Toxins (2019) 11:98. doi: 10.3390/toxins11020098 PMC6409564 30736358

[192] Roberts A Harris H Smith M Giles J Polak O Buckley A. A novel, orally delivered antibody therapy and its potential to prevent clostridioides difficile infection in pre-clinical models. Front Microbiol (2020) 11. doi: 10.3389/fmicb.2020.578903 PMC7537341 33072047

[193] Chiari E Weiss W Simon M Kiessig S Pulse M Brown S. Oral immunotherapy with human secretory IgA improves survival in the hamster model of clostridioides difficile infection. J Infect Dis 224(8):1394–97(2021). doi: 10.1093/infdis/jiab087 PMC8557658 33588433

[194] Lyerly D Phelps C Toth J Wilkins T. Characterization of toxins a and b of clostridium difficile with monoclonal antibodies. Infect Immunity (1986) 54:70–6. doi: 10.1128/iai.54.1.70-76.1986 PMC260118 2428753

[195] Frey S Wilkins T. Localization of two epitopes recognized by monoclonal antibody PCG-4 on clostridium difficile toxin a. Infect Immunity (1992) 60:2488–92. doi: 10.1128/iai.60.6.2488-2492.1992 PMC257186 1375199

[196] Lyerly D Phelps C Wilkins T. Monoclonal and specific polyclonal antibodies for immunoassay of clostridium difficile toxin a. J Clin Microbiol (1985) 21:12–4. doi: 10.1128/jcm.21.1.12-14.1985 PMC271570 3968199

[197] Modi N Gulati N Monaghan T Robins A Sewell H Mahida Y. Differential binding and internalization of clostridium difficile toxin a by human peripheral blood monocytes, neutrophils and lymphocytes. Scandinavian J Immunol (2011) 74:264–71. doi: 10.1111/j.1365-3083.2011.02578.x 21595735

[198] Kamiya S Yamakawa K Meng X Ogura H Nakamura S. Production of monoclonal antibody to clostridium difficile toxin a which neutralises enterotoxicity but not haemagglutination activity. FEMS Microbiol Letters (1991) 65:311–5. doi: 10.1111/j.1574-6968.1991.tb04778.x 1916231

[199] Corthier G Muller MC Wilkins TD Lyerly D L'Haridon R. Protection against experimental pseudomembranous colitis in gnotobiotic mice by use of monoclonal antibodies against clostridium difficile toxin a. Infect Immun (1991) 59:1192–5. doi: 10.1128/iai.59.3.1192-1195.1991 PMC258389 1900059

[200] Demarest S Hariharan M Elia M Salbato J Jin P Bird C. Neutralization of clostridium difficile toxin a using antibody combinations. MAbs (2010) 2:190–8. doi: 10.4161/mabs.2.2.11220 PMC2840238 20150758

[201] He X Sun X Wang J-F Wang X Zhang Q Tzipori S. Antibody-enhanced, fc gamma receptor-mediated endocytosis of clostridium difficile toxin a. Infect Immun (2009) 77:2294–303. doi: 10.1128/IAI.01577-08 PMC2687358 19307220

[202] Steele J Mukherjee J Parry N Tzipori S. Antibody against TcdB, but not TcdA, prevents development of gastrointestinal and systemic clostridium difficile disease. J Infect Dis (2012) 207:323–330. doi: 10.1093/infdis/jis669 PMC3532825 23125448

[203] Zhang C Jin K Xiao Y Cheng Y Huang Z Wang S. Potent monoclonal antibodies against clostridium difficile toxin a elicited by DNA immunization. Hum Vaccines Immunotherapeutics (2013) 9:2157–2164. doi: 10.4161/hv.25656 PMC3906400 23851482

[204] Babcock G Broering T Hernandez H Mandell R Donahue K Boatright N. Human monoclonal antibodies directed against toxins a and b prevent clostridium difficile-induced mortality in hamsters. Infect Immun (2006) 74:6339–47. doi: 10.1128/IAI.00982-06 PMC1695490 16966409

[205] Taylor C Tummala S Molrine D Davidson L Farrell R Lembo A. Open-label, dose escalation phase I study in healthy volunteers to evaluate the safety and pharmacokinetics of a human monoclonal antibody to clostridium difficile toxin a. Vaccine (2008) 26:3404–9. doi: 10.1016/j.vaccine.2008.04.042 PMC2628753 18502001

[206] Leav B Blair B Leney M Knauber M Reilly Csikesz C Lowy I. Serum anti-toxin b antibody correlates with protection from recurrent clostridium difficile infection (CDI). Vaccine (2009) 28:965–9. doi: 10.1016/j.vaccine.2009.10.144 19941990

[207] Yang Z Ramsey J Hamza T Zhang Y li S Yfantis H. Mechanisms of protection against clostridium difficile infection by the monoclonal antitoxin antibodies actoxumab and bezlotoxumab. Infect Immun (2014) 83:822–31. doi: 10.1128/IAI.02897-14 PMC4294251 25486992

[208] Anosova N Cole L Li L Zhang J Brown A Mundle S. A combination of three fully-human toxin a- and toxin b-specific monoclonal antibodies protects against challenge with highly virulent epidemic strains of c. difficile in the hamster model. Clin Vaccine Immunol CVI (2015) 22:711–725. doi: 10.1128/CVI.00763-14 PMC4478530 25924765

[209] Marozsan A Ma D Nagashima K Kennedy B Kang K Arrigale R. Protection against clostridium difficile infection with broadly neutralizing antitoxin monoclonal antibodies. J Infect Dis (2012) 206:706–13. doi: 10.1093/infdis/jis416 PMC3491748 22732923

[210] Koon H Shih D Hing T Yoo J Ho S Chen X. Human monoclonal antibodies against clostridium difficile toxins a and b inhibit inflammatory and histologic responses to the toxins in human colon and peripheral blood monocytes. Antimicrobial Agents Chemotherapy (2013) 57:3214–3223. doi: 10.1128/AAC.02633-12 PMC3697353 23629713

[211] Qiu H Cassan R Johnstone D Han X Joyee AG McQuoid M. Novel clostridium difficile anti-toxin (TcdA and TcdB) humanized monoclonal antibodies demonstrate *In vitro* neutralization across a broad spectrum of clinical strains and *In vivo* potency in a hamster spore challenge model. PLoS One (2016) 11(6):e0157970–e. doi: 10.1371/journal.pone.0157970 PMC4919053 27336843

[212] Deng X Nesbit L Morrow K. Recombinant single-chain variable fragment antibodies directed against clostridium difficile toxin b produced by use of an optimized phage display system. Clin Diagn Lab Immunol (2003) 10:587–95. doi: 10.1128/CDLI.10.4.587-595.2003 PMC164272 12853390

[213] Hussack G Arbabi Ghahroudi M Faassen H Songer J Ng K Mackenzie R. Neutralization of clostridium difficile toxin a with single-domain antibodies targeting the cell receptor binding domain. J Biol Chem (2011) 286:8961–76. doi: 10.1074/jbc.M110.198754 PMC3058971 21216961

[214] Hussack G Hirama T Ding W Mackenzie R Tanha J. Engineered single-domain antibodies with high protease resistance and thermal stability. PLoS One (2011) 6(11):e28218–e. doi: 10.1371/journal.pone.0028218 PMC3227653 22140551

[215] Murase T Eugenio L Schorr M Hussack G Tanha J Kitova E. Structural basis for antibody recognition in the receptor-binding domains of toxins a and b from clostridium difficile. J Biol Chem (2013) 289:2331–2343. doi: 10.1074/jbc.M113.505917 PMC3900976 24311789

[216] Shkoporov A Khokhlova E Savochkin KA Kafarskaia L Efimov BA. Production of biologically active scFv and VHH antibody fragments in. FEMS Microbiol Lett (2015) 362. doi: 10.1093/femsle/fnv083 25994292

[217] Yang Z Schmidt D Liu W li S Shi L Sheng J. A novel multivalent, single-domain antibody targeting TcdA and TcdB prevents fulminant clostridium difficile infection in mice. J Infect Dis (2014) 210:964–972. doi: 10.1093/infdis/jiu196 PMC4192054 24683195

[218] Andersen KK Strokappe NM Hultberg A Truusalu K Smidt I Mikelsaar R-H. Neutralization of clostridium difficile toxin b mediated by engineered lactobacilli that produce single-domain antibodies. Infect Immun (2015) 84(2):395–406. doi: 10.1128/IAI.00870-15 PMC4730582 26573738

[219] Kandalaft H Hussack G Aubry A Faassen H Guan Y Arbabi Ghahroudi M. Targeting surface-layer proteins with single-domain antibodies: a potential therapeutic approach against clostridium difficile-associated disease. Appl Microbiol Biotechnol (2015) 99:8549–8562. doi: 10.1007/s00253-015-6594-1 PMC4768215 25936376

[220] Nazari Shirvan A Aitken R. Isolation of recombinant antibodies directed against surface proteins of clostridium difficile. Braz J Microbiol (2016) 47:394–402. doi: 10.1016/j.bjm.2016.01.017 PMC4874623 26991284

[221] Fuehner V Heine P Helmsing S Goy S Heidepriem J Loeffler F. Development of neutralizing and non-neutralizing antibodies targeting known and novel epitopes of TcdB of clostridioides difficile. Front Microbiol (2018) 9:2908. doi: 10.3389/fmicb.2018.02908 30574127 PMC6291526

[222] Maynard-Smith M Ahern H McGlashan J Nugent P Ling R Denton H. Recombinant antigens based on toxins a and b of clostridium difficile that evoke a potent toxin-neutralising immune response. Vaccine (2013) 32:700–705. doi: 10.1016/j.vaccine.2013.11.099 PMC3969267 24342251

[223] Huang J-H Wu C-W Lien S-P Leng C-H Hsiao K-N Liu S-J. Recombinant lipoprotein-based vaccine candidates against c. difficile infections. J Biomed Sci (2015) 22:65. doi: 10.1186/s12929-015-0171-x 26245825 PMC4527207

[224] Bézay N Ayad A Dubischar K Firbas C Hochreiter R Kiermayr S. Safety, immunogenicity and dose response of VLA84, a new vaccine candidate against clostridium difficile, in healthy volunteers. Vaccine (2016) 34:2585–2592. doi: 10.1016/j.vaccine.2016.03.098 27079932

[225] Luo D Liu X Xing L Sun Y Huang J Zhang L. Immunogenicity and protection from receptor-binding domains of toxins as potential vaccine candidates for clostridium difficile. Vaccines (2019) 7:180. doi: 10.3390/vaccines7040180 PMC6963439 31717334

[226] Graham B Ambrosino D. History of passive antibody administration for prevention and treatment of infectious diseases. Curr Opin HIV AIDS (2015) 10:129–134. doi: 10.1097/COH.0000000000000154 PMC4437582 25760933

[227] Sponseller J Steele J Schmidt D Kim HB Beamer G Sun X. Hyperimmune bovine colostrum as a novel therapy to combat clostridium difficile infection. J Infect Dis (2014) 211:1334–1341. doi: 10.1093/infdis/jiu605 PMC4447838 25381448

[228] Kyne L Warny M Qamar A Kelly CP. Asymptomatic carriage of clostridium difficile and serum levels of IgG antibody against toxin a. New Engl J Med (2000) 342(6):390–7. doi: 10.1056/NEJM200002103420604 10666429

[229] Beales I. Intravenous immunoglobulin for recurrent clostridium difficile diarrhoea. Gut (2002) 51:456. doi: 10.1136/gut.51.3.456 PMC1773353 12171976

[230] Shah N Shaaban H Spira R Slim J Boghossian J. Intravenous immunoglobulin in the treatment of severe clostridium difficile colitis. J Global Infect Diseases (2014) 6(2):82–5. doi: 10.4103/0974-777X.132053 PMC4049046 24926170

[231] Shahani L Koirala J. Use of intravenous immunoglobulin in severe clostridium difficile-associated diarrhea. Hosp Pract (1995) (2015) 43:1–4. doi: 10.1080/21548331.2015.1071636 26189489

[232] Numan S Veldkamp P Kuijper E Berg R Dissel J. Clostridium difficile-associated diarrhoea: Bovine anti-clostridium difficile whey protein to help aid the prevention of relapses. Gut (2007) 56:888–9. doi: 10.1136/gut.2006.119016 PMC1954844 17519495

[233] Kyne L Kelly CN. Prospects for a vaccine for clostridium difficile. BioDrugs Clin Immunotherapeutics biopharmaceuticals Gene Ther (1998) 10:173–81. doi: 10.2165/00063030-199810030-00001 18020593

[234] Wilcox M Gerding D Poxton I Kelly C Nathan R Cornely O. Bezlotoxumab alone and with actoxumab for prevention of recurrent clostridium difficile infection in patients on standard of care antibiotics: Integrated results of 2 phase 3 studies (MODIFY I and MODIFY II). Open Forum Infect Dis (2015) 2(suppl_1):305–317. doi: 10.1093/ofid/ofv131.06

[235] Navalkele BD Chopra T. Bezlotoxumab: an emerging monoclonal antibody therapy for prevention of recurrent clostridium difficile infection. Biologics Targets Ther (2018) 12:11–21. doi: 10.2147/BTT.S127099 PMC5779312 29403263

[236] van Prehn J Reigadas E Vogelzang EH Bouza E Hristea A Guery B. European Society of clinical microbiology and infectious diseases: 2021 update on the treatment guidance document for clostridioides difficile infection in adults. Clin Microbiol Infect (2021) 27:S1–S21. doi: 10.1016/j.cmi.2021.09.038 34678515

[237] Lam SW Neuner EA Fraser TG Delgado D Chalfin DB. Cost-effectiveness of three different strategies for the treatment of first recurrent clostridium difficile infection diagnosed in a community setting. Infect Control Hosp Epidemiol (2018) 39(8):924–30. doi: 10.1017/ice.2018.139 29961435

[238] Gupta A Ananthakrishnan AN. Economic burden and cost-effectiveness of therapies for clostridiodes difficile infection: a narrative review. Therap Adv Gastroenterol (2021) 14:17562848211018654. doi: 10.1177/17562848211018654 PMC8170348 34104214

[239] Dubberke ER Gerding DN Kelly CP Garey KW Rahav G Mosley A. Efficacy of bezlotoxumab in participants receiving metronidazole, vancomycin, or fidaxomicin for treatment of clostridioides (Clostridium) difficile infection. Open Forum Infect Dis (2020) 7(6):ofaa157. doi: 10.1093/ofid/ofaa157 32523972 PMC7264839

[240] Prabhu VS Dubberke ER Dorr MB Elbasha E Cossrow N Jiang Y. Cost-effectiveness of bezlotoxumab compared with placebo for the prevention of recurrent clostridium difficile infection. Clin Infect Dis (2018) 66(3):355–62. doi: 10.1093/cid/cix809 29106516

[241] Oksi J Aalto A Säilä P Partanen T Anttila VJ Mattila E. Real-world efficacy of bezlotoxumab for prevention of recurrent clostridium difficile infection: a retrospective study of 46 patients in five university hospitals in Finland. Eur J Clin Microbiol Infect Dis (2019) 38:1947–52. doi: 10.1007/s10096-019-03630-y PMC6778539 31359254

[242] Gerding D Kelly C Rahav G Lee C Dubberke E Kumar P. Bezlotoxumab for prevention of recurrent c. difficile infection in patients at increased risk for recurrence. Clin Infect Dis An Off Publ Infect Dis Soc America (2018) 67:649–56. doi: 10.1093/cid/ciy171 PMC6093994 29538686

[243] Alonso C Mahoney M. Bezlotoxumab for the prevention of clostridium difficile infection: a review of current evidence and safety profile. Infect Drug Resistance (2018) 12:1–9. doi: 10.2147/IDR.S159957 PMC6301304 30588042

[244] Corraliza-Gorjón I Somovilla-Crespo B Santamaria S Garcia-Sanz JA Kremer L. New strategies using antibody combinations to increase cancer treatment effectiveness. Front Immunol (2017) 8:1804. doi: 10.3389/fimmu.2017.01804 29312320 PMC5742572

[245] Powers D Amersdorfer P Poul M-A Nielsen U Shalaby MR Adams G. Expression of single-chain fv-fc fusions in pichia pastoris. J Immunol Methods (2001) 251:123–35. doi: 10.1016/S0022-1759(00)00290-8 11292488

[246] Zhang J Mackenzie R Durocher Y. Production of chimeric heavy-chain antibodies. Methods Mol Biol (2009) 525:323–36. doi: 10.1007/978-1-59745-554-1_17 19252853

[247] Stanfield R Dooley H Flajnik M Wilson I Stanfield RL Dooley H. Crystal structure of a shark single-domain antibody V region in complex with lysozyme. Science (2004) 305:1770–3. doi: 10.1126/science.1101148 15319492

[248] Hussack G Tanha J Heidebrecht H-J Weiss WJ Pulse M Lange A. Toxin-specific antibodies for the treatment of clostridium difficile: Current status and future perspectives. Toxins (2010) 2(5):998–1018. doi: 10.3390/toxins2050998 PMC3153223 22069622

[249] Wemmer S Mashau C Fehrsen J Wyngaardt W Plessis D. Chicken scFvs and bivalent scFv-CH fusions directed against HSP65 of mycobacterium bovis. Biologicals J Int Assoc Biol Standardization (2010) 38:407–14. doi: 10.1016/j.biologicals.2010.02.002 20299243

[250] Liu H Saxena A Sidhu S Wu D. Fc engineering for developing therapeutic bispecific antibodies and novel scaffolds. Front Immunol (2017) 8. doi: 10.3389/fimmu.2017.00038 PMC5266686 28184223

[251] Adams H Brummelhuis W Maassen B Egmond N Khattabi M Detmers F. Specific immuno capturing of the staphylococcal superantigen toxic-shock syndrome toxin-1 in plasma. Biotechnol Bioengineering (2009) 104:143–51. doi: 10.1002/bit.22365 19475676

[252] Tremblay J Mukherjee J Leysath C Debatis M Ofori K Baldwin K. Single VHH-based toxin-neutralizing agent and an effector antibody protect mice against challenge with shiga toxins 1 and 2. A Infect Immun (2013) 81:4595–4603. doi: 10.1128/IAI.01033-13 PMC3837998 24082082

[253] Unger M Eichhoff A Schumacher L Strysio M Menzel S Schwan C. Selection of nanobodies that block the enzymatic and cytotoxic activities of the binary clostridium difficile toxin CDT. Sci Rep (2015) 5. doi: 10.1038/srep07850 PMC4297958 25597743

[254] Castoldi R Jucknischke U Pradel L Arnold E Klein C Scheiblich S. Molecular characterization of novel trispecific ErbB-cMet-IGF1R antibodies and their antigen-binding properties. Protein Engineering Design Selection PEDS (2012) 25:551–60. doi: 10.1093/protein/gzs048 PMC3449402 22936109

[255] Jachimowicz R Borchmann S Rothe A. Multi-specific antibodies for cancer immunotherapy. BioDrugs Clin Immunotherapeutics biopharmaceuticals Gene Ther (2014) 28:331–343. doi: 10.1007/s40259-014-0091-4 24638872

[256] Spiess C Zhai Q Carter P. Alternative molecular formats and therapeutic applications for bispecific antibodies. Mol Immunol (2015) 310:95–106. doi: 10.1016/j.molimm.2015.01.003 25637431

[257] Byrne H Conroy P Whisstock J O'Kennedy R. A tale of two specificities: Bispecific antibodies for therapeutic and diagnostic applications. Trends Biotechnol (2013) 31:621–632. doi: 10.1016/j.tibtech.2013.08.007 PMC7114091 24094861

[258] Fan G Wang Z Hao M Li J. Bispecific antibodies and their applications. J Hematol Oncol (2015) 8(1):130. doi: 10.1186/s13045-015-0227-0 26692321 PMC4687327

[259] Hussack G Ryan S Faassen H Rossotti M Mackenzie C Tanha J. Neutralization of clostridium difficile toxin b with VHH-fc fusions targeting the delivery and CROPs domains. PLoS One (2018) 13:e0208978. doi: 10.1371/journal.pone.0208978 30540857 PMC6291252

[260] Sulea T Hussack G Ryan S Tanha J Purisima EO. Application of assisted design of antibody and protein therapeutics (ADAPT) improves efficacy of a clostridium difficile toxin a single-domain antibody. Sci Rep (2018) 8(1):2260. doi: 10.1038/s41598-018-20599-4 29396522 PMC5797146

[261] Shkoporov AN Khokhlova EV Savochkin KA Kafarskaia LI Efimov BA. Production of biologically active scFv and VHH antibody fragments in bifidobacterium longum. FEMS Microbiol Lett (2015) 362(12):fnv083. doi: 10.1093/femsle/fnv083 25994292

[262] Chen K Zhu Y Zhang Y Hamza T Yu H Saint Fleur A. A probiotic yeast-based immunotherapy against clostridioides difficile infection. Sci Transl Med (2020) 12(567):eaax4905. doi: 10.1126/scitranslmed.aax4905 33115949 PMC7692727

[263] Eckert C Emirian A Le Monnier A Cathala L Montclos H Goret J. Prevalence and pathogenicity of binary toxin–positive clostridium difficile strains that do not produce toxins a and b. New Microbes New Infections (2015) 3:12–7. doi: 10.1016/j.nmni.2014.10.003 PMC4337936 25755885

[264] Calabi E Calabi F Phillips A Fairweather N. Binding of clostridium difficile surface layer proteins to gastrointestinal tissues. Infect Immunity (2002) 70:5770–8. doi: 10.1128/IAI.70.10.5770-5778.2002 PMC128314 12228307

[265] Merrigan M Venugopal A Roxas J Anwar F Mallozzi M Roxas B. (SlpA) is a major contributor to host-cell adherence of clostridium difficile. PLoS One (2013) 8:e78404. doi: 10.1371/journal.pone.0078404 24265687 PMC3827033

[266] Stevenson E Minton N Kuehne S. The role of flagella in clostridium difficile pathogenicity. Trends Microbiol (2015) 23:275–282. doi: 10.1016/j.tim.2015.01.004 25659185

[267] Pantaléon V Soavelomandroso A Bouttier S Briandet R Roxas B Chu M. The clostridium difficile protease Cwp84 modulates both biofilm formation and cell-surface properties. PLoS One (2015) 10. 10.1371/journal.pone.0124971 PMC4414356 25922949

[268] Péchiné S Janoir C Boureau H Gleizes A Tsapis N Hoys S. Diminished intestinal colonization by clostridium difficile and immune response in mice after mucosal immunization with surface proteins of clostridium difficile. Vaccine (2007) 25:3946–54. doi: 10.1016/j.vaccine.2007.02.055 17433506

[269] Eidhin D O'Brien J McCabe M Athie V Kelleher D. Active immunization of hamsters against clostridium difficile infection using surface-layer protein. FEMS Immunol Med Microbiol (2008) 52:207–18. doi: 10.1111/j.1574-695X.2007.00363.x 18093141

[270] Adawi A Bisignano C Genovese T Filocamo A Khouri-Assi C Neville A. *In vitro* and *in vivo* properties of a fully human IgG1 monoclonal antibody that combats multidrug resistant pseudomonas aeruginosa. Int J Mol Med (2012) 30(3):455–64. doi: 10.3892/ijmm.2012.1040 PMC3573743 22735858

[271] Anton P O'Brien M Kokkotou E Eisenstein B Michaelis A Rothstein D. Rifalazil treats and prevents relapse of clostridium difficile-associated diarrhea in hamsters. Antimicrob Agents Chemother (2004) 48:3975–9. doi: 10.1128/AAC.48.10.3975-3979.2004 PMC521872 15388461

[272] Pear S Williamson T Bettin K Gerding D Galgiani J. Decrease in nosocomial clostridium difficile-associated diarrhea by restricting clindamycin use. Ann Internal Med (1994) 120:272–7. doi: 10.7326/0003-4819-120-4-199402150-00003 8080497

[273] Bockstaele F Holz J-B Revets H. The development of nanobodies for therapeutic applications. Curr Opin Investigational Drugs (2009) 10:1212–24. 19876789

[274] Laroui H Dalmasso G Nguyen H Yan Y Sitaraman S Merlin D. Drug-loaded nanoparticles targeted to the colon with polysaccharide hydrogel reduce colitis in a mouse model. Gastroenterology (2009) 138:843–53.e1. doi: 10.1053/j.gastro.2009.11.003 19909746

[275] Brandt L Aroniadis O Mellow M Kanatzar A Kelly C Park T. Long-term follow-up of colonoscopic fecal microbiota transplant for recurrent clostridium difficile infection. Am J Gastroenterol (2012) 107:1079–87. doi: 10.1038/ajg.2012.60 22450732

[276] Azimirad M Yadegar A Gholami F Shahrokh S Asadzadeh Aghdaei H Ianiro G. Treatment of recurrent clostridioides difficile infection using fecal microbiota transplantation in Iranian patients with underlying inflammatory bowel disease. J Inflammation Res (2020) 13:563–70. doi: 10.2147/JIR.S265520 PMC7509309 32982371

[277] Azimirad M Jo Y Kim MS Jeong M Shahrokh S Asadzadeh Aghdaei H. Alterations and prediction of functional profiles of gut microbiota after fecal microbiota transplantation for Iranian recurrent clostridioides difficile infection with underlying inflammatory bowel disease: A pilot study. J Inflammation Res (2022) 15:105–16. doi: 10.2147/JIR.S338212 PMC8747792 35023946

[278] Roovers R Vosjan M Laeremans T Khoulati R de Bruin R Ferguson K. A biparatopic anti-EGFR nanobody efficiently inhibits solid tumour growth. Int J Cancer J Int Du Cancer (2011) 129:2013–24. doi: 10.1002/ijc.26145 PMC4197845 21520037

[279] Bannas P Hambach J Koch-Nolte F. Nanobodies and nanobody-based human heavy chain antibodies as antitumor therapeutics. Front Immunol (2017) 8:1603. doi: 10.3389/fimmu.2017.01603 29213270 PMC5702627

[280] Godakova S Noskov A Vinogradova I Ugriumova G Solovyev A Esmagambetov I. Camelid VHHs fused to human fc fragments provide long term protection against botulinum neurotoxin a in mice. Toxins (2019) 11. doi: 10.3390/toxins11080464 PMC6723419 31394847

[281] Pelat T Hust M Laffly E Condemine F Bottex C Vidal D. High-affinity, human antibody-like antibody fragment (single-chain variable fragment) neutralizing the lethal factor (LF) of bacillus anthracis by inhibiting protective antigen-LF complex formation. Antimicrobial Agents Chemo (2007) 51(8):2758–64. doi: 10.1128/AAC.01528-06 PMC1932538 17517846

[282] Kim D Hussack G Kandalaft H Tanha J. Mutational approaches to improve the biophysical properties of human single-domain antibodies. Biochim Biophys Acta (2014) 1844:1983–2001. doi: 10.1016/j.bbapap.2014.07.008 25065345

[283] Andersen KK Marcotte H Álvarez B Boyaka PN Hammarström L. *In situ* gastrointestinal protection against anthrax edema toxin by single-chain antibody fragment producing lactobacilli. BMC Biotechnol (2011) 11:126. doi: 10.1186/1472-6750-11-126 22185669 PMC3295704

[284] Vandenbroucke K de Haard H Beirnaert E Dreier T Lauwereys M Huyck L. Orally administered l. lactis secreting an anti-TNF nanobody demonstrate efficacy in chronic colitis. Mucosal Immunol (2009) 3:49–56. doi: 10.1038/mi.2009.116 19794409

[285] Martín M Pant N Ladero V Günaydın G Krogh Andersen K Álvarez B. Integrative expression system for delivery of antibody fragments by lactobacilli. Appl Environ Microbiol (2011) 77:2174–9. doi: 10.1128/AEM.02690-10 PMC3067310 21257814

[286] Pant N Marcotte H Hermans P Bezemer S Frenken L Johansen K. Lactobacilli producing bispecific llama-derived anti-rotavirus proteins *in vivo* for rotavirus-induced diarrhea. Future Microbiol (2011) 6:583–93. doi: 10.2217/fmb.11.32 21585264

[287] Günaydın G Álvarez B Lin Y Hammarström L Marcotte H. Co-Expression of anti-rotavirus proteins (Llama VHH antibody fragments) in lactobacillus: Development and functionality of vectors containing two expression cassettes in tandem. PLoS One (2014) 9:e96409. doi: 10.1371/journal.pone.0096409 24781086 PMC4004553

